# State of the Art of Anthocyanins: Antioxidant Activity, Sources, Bioavailability, and Therapeutic Effect in Human Health

**DOI:** 10.3390/antiox9050451

**Published:** 2020-05-23

**Authors:** Noelia Tena, Julia Martín, Agustín G. Asuero

**Affiliations:** 1 Departamento de Química Analítica, Facultad de Farmacia, Universidad de Sevilla, Prof. García González 2, E-41012 Sevilla, Spain; asuero@us.es; 2 Departamento de Química Analítica, Escuela Politécnica Superior, Universidad de Sevilla, C/Virgen de África 7, E-41011 Sevilla, Spain; jbueno@us.es

**Keywords:** anthocyanins, antioxidant activity, anthocyanin content, bioavailability, encapsulation, therapeutic effects

## Abstract

The antioxidant activity of anthocyanins in food is well known. Numerous antioxidant assays have been proposed to measure the capacity of anthocyanins to prevent the oxidation process that naturally occurs. Different solvents, temperatures, and pH levels are applied in each assay, and these factors should be taken into account in order to obtain useful and reproducible results. The concentration and the structure of these compounds are directly related to their antioxidant capacity and their environment. However, the effectiveness of the anthocyanin ingestion against diseases is also influenced by its bioavailability. Novel methodologies that simulate the digestion process have been developed in order to facilitate the current knowledge of anthocyanins bioavailability. Studies highlight the potential synergy effect between parent compounds and their derivatives (metabolites, conjugated products, and microbe-generated metabolites). The aim of this review is to provide an overview of advantages and disadvantages of the most common methods to determine the antioxidant activity of anthocyanins, chemical structure, and concentration of these compounds in different edible fruits, vegetables, and plants; their bioavailability after intake; as well as the main therapeutic effect described in the scientific literature.

## 1. Introduction

In recent years, the interest in plants and food containing antioxidant properties has increased. The chemical compounds present in vegetables and fruits with these capacities are: vitamins C and E, carotenoids, and flavonoids. The anthocyanins, which are the most important group of flavonoids in plants, are pigments with a flavylium cation (AH^+^) structure that act as acid. This structure is directly related to its antioxidant activity. Most of the functional properties and the sensory quality of the anthocyanins can be explained by their chemical reactivity. The structures and properties of anthocyanins are dependent on different factors such as pH, temperature, and solvents which should be controlled to carry out antioxidant activity studies of these compounds [1,2,3,4,5,6,7,8,9].

Free radicals, reactive oxygen species (ROS), and/or reactive nitrogen species (RNS) are required for the proper performance of the human body and its organs. These radicals are on-balance by a redox homeostasis in our body. However, the body may be occasionally affected by an oxidative stress resulting from an off-balance state. This stress is important in the development of chronic degenerative diseases including coronary heart disease, cancer, and aging [2]. Anthocyanins have been described as compounds that prevent or inhibit, the oxidation by scavenging free radicals and reducing the oxidative stress. On a regular basis, anthocyanins act as H-atom donator or as single electron transfer. Different methods of analysis based on both mechanisms have been proposed to determine the antioxidant activity of anthocyanins. The antioxidant activity of these compounds depends on their total concentration, structure, and environment. A literature compilation about the concentrations of the most common anthocyanins in different foods is presented in this review in order to have an overview of the different sources of anthocyanins.

The beneficial properties attributed to the dietary ingestion of anthocyanin-rich foods (eye health, cardiovascular diseases, antiobesity, antidiabetic, antimicrobial, anticancer or neuroprotective effect) have been deeply documented in studies carried out with experimental models. These health benefits contrast with the apparent small portion (<1–2%) of these compounds absorbed by our organism [3,4,5]. During the digestion process, anthocyanins undergo to an intense variation in pH that together with the enzymatic and bacterial action can cause the hydrolysis and transformation of anthocyanins into metabolites, conjugated products, or simpler phenolic compounds [7,10,11,12]. The question is: How can anthocyanins be so influential in health? Are anthocyanins the only responsible of their beneficial effects? Last scientific developments highlight the potential synergy effect between parent compounds, metabolites (phases I and II), conjugated products, and microbe-generated metabolites to explain those biological events [4,11,12,13].

Due to their particular physicochemical features, bioavailability of anthocyanins is very difficult to assess. The first studies were performed analyzing blood and urine to determine the anthocyanin concentration levels after the ingestion of foods rich in anthocyanins [14,15,16]. However, the low absorption percentage obtained led to in vitro assays (mostly using cell culture systems) in order to facilitate the knowledge of their biochemical and chemical changes as well as the influence of the digestion steps. Last studies have emphasized the key role of the microbiota in the transformation of anthocyanins, which is not considered in in vitro assays but it is still poorly considered in in vivo and ex vivo studies [14].

This review aims to highlight some aspects regarding the antioxidant activity of anthocyanins and their bioavailability after intake. The first part includes an exposition of the most common antioxidant bioassays used to determine in vitro the antioxidant activity of anthocyanins, being the advantages and disadvantages of each bioassay identified. Afterwards, the effect of the chemical structure and the environment in the ability of the anthocyanins to prevent oxidation is discussed and presented together with information about different sources and range of concentration of these compounds in food. The second part of the manuscript exposes the bioavailability and metabolism of anthocyanins as well as a summary including the main therapeutic effects of anthocyanins on different diseases.

## 2. Antioxidant Bioassays for Anthocyanins

Numerous antioxidant assays have been proposed to measure the ability of anthocyanins to prevent the oxidation process that naturally occurs. Depending on the source of the anthocyanins and their nature, in most of the cases, an extraction step before carrying out the antioxidant bioassay is needed. The extraction process is a critical step in the determination of the antioxidant activity bioassay, presenting a challenge due to the low stability of anthocyanins after extraction and their tendency to remain bound to the matrix of the sample. Multiple alternatives have been proposed in literature for this procedure [17]. Results show that temperature, pH, solvent system, solvent-to-solid ratio, and number of extractions are factors that play an important role in the extraction efficiency and that should be optimized for each sample [18,19]. Once the anthocyanins are in liquid solution, the antioxidant activity could be determined by different bioassays. In general, two different mechanisms can be used to explain the antioxidant activity of anthocyanins: Hydrogen atom donator (HAT) and single-electron transfer (SET). In HAT mechanism, the free radical R^•^ removes a hydrogen atom from the antioxidant (AH^+^) converting the free radical to a more stable product. In the SET mechanism, the antioxidant (AH^+^) donates an electron to the free radical reducing the oxidized intermediates into the stable form [20]. However, the difficulty in distinguishing between HAT and SET reactions is high. In most situations, these two reactions take place simultaneously, and the mechanism of the reaction is determined by the antioxidant structure, solubility, the partition coefficient, and solvent polarity [21]. Different antioxidant bioassays have been commonly used in the literature to determine the antioxidant activity of anthocyanins. A summary of the most common antioxidant bioassays used for anthocyanins is shown in Table 1.

Each one of these methods provides information about the antioxidant activity of the anthocyanins under specific conditions. In order to have more information about the reaction mechanism, a more comprehensive description of the procedure followed in each one of these bioassays are presented together with their main advantages and disadvantages.

### 2.1. DPPH (Diphenyl-1-Picrylhydrazyl) Assay

DPPH assay is a spectrophotometric method that can be applied for both solid and liquid samples, not being specific for any particular antioxidant. Thus, it can be applied to determine the total antioxidant capacity of the sample. This assay is based on the ability of the free radical (DPPH^•^) to react with hydrogen donor (AH^+^). The free radical presents an intense abortion in the UV–vis spectral region at 515 nm, the absorbance at 515 nm decreases when the free radical is reduced.

The antioxidant activity of anthocyanins for DPPH has been assessed by several authors [22,23,24,25,26]. Reliable results can be obtained with this method in a fast and simple manner. Currently, a colorimetric alternative has been proposed to extend the application of this assay where a spectrophotometer is not available [27]. Furthermore, as the free radical is stable its production is not necessary every time that an analysis is carried out. The main disadvantages could be the complexity of the analysis, as it turns more complex when other compounds present in the sample absorb also at 515 nm and many antioxidants are inert to DPPH.

### 2.2. ORAC Assay (Oxygen Radical Absorbance Capacity)

ORAC assay is a fluorescence method that combines the sample (AH^+^) with a fluorescent compound, protein phycoerthrin (β-PE) and with a generator of free radicals, 2,2′-azobis(2-amidino-propane) dihydrochloride (AAPH). This assay is based on the loss of the fluorescent compound when it is oxidized by the generated free radicals. When antioxidants (AH^+^) are involved in the reaction, the fluorescent compound is protected from oxidative degradation. Hence, the fluorescence signal remains. The fluorescence signal is monitored during 1 h at λem = 565 nm and λex = 540 nm respectively. The antioxidant activity of anthocyanins has been assessed by ORAC assay by several authors [26,28,29,30]. This is considered a good method to determine the antioxidant capacity of hydrophilic and hydrophobic samples, as it is easily adaptive to different samples changing the generator of free radicals. One disadvantage is the non-specificity of the fluorescence compounds that can react with the sample losing fluorescence even without the addition of a free radical generator.

### 2.3. TRAP Assay (Total Peroxyl Radical Trapping Antioxidant Parameter)

TRAP assay is a chemiluminescence method that consists of the following components: (i) thermolabile azo-radical initiator (e.g., 2,2′-azobis(2-amidopropane) hydrochloride (ABAP)), which produces radicals (R^•^) that react rapidly with O_2_ to give a peroxyl radicals (ROO^•^); (ii) oxidizable compounds with chemiluminescence properties to monitor the reaction progress (e.g., Luminol); and (iii) the sample with the antioxidant properties (AH^+^).

The antioxidant activity of anthocyanins has been assessed by TRAP assay by several authors [31,32,33,34]. This assay is sensitive to all known chain-breaking antioxidants. However, an important disadvantage to highlight is the difficulty comparing the results between laboratories due to the amount of different end points that can be used. It is relatively complex method, time-consuming, and requires a high degree of experience. Furthermore, and such as ORAC assay, the oxidizable compounds are non-specific and other non-radical chain reaction could occur.

### 2.4. FCT (Ferric Thiocyanate) Assay

The FTC assay is a spectrophotometric method that consists of the oxidation of ferrous chloride to ferric ion by reacting with peroxide. The peroxides are formed during the reaction that takes place when the sample is mixed with ethanol, water, phosphate buffer solution (pH = 7), and linoleic acid. Then, ferrous chloride in hydrochloric acid is added to the reaction and the ferric ion formed is combined with ammonium thiocyanate producing ferric thiocyanate, which is red. The absorbance of the sample is measured at 500 nm until the maximum value is reached.

This assay is used to measure the amount of peroxide produced during the initial stages of oxidation. In the case of the anthocyanins this method has been applied to determine the antioxidant activity avoiding the peroxidation of polyunsaturated fatty acid [35]. This assay is simple and reproducible. Nevertheless, results are not reliable in the case that compounds within the sample absorb around 500 nm. This drawback is common not only in this method, but in other spectrophotometric assays.

### 2.5. FRAP (Ferric Reducing Antioxidant Power) Assay

FRAP assay is a colorimetric method that can be employed for the determination of the total antioxidant activity of anthocyanins. It is based on the reduction of complexes of 2,4,6-tripyridyl-s-triazine (TPTZ) with ferric chloride hexahydrate (FeCl_3_·6H_2_O) under acidic conditions. The solution turns slightly brownish, forming blue ferrous complexes once the reduction is completed. The absorbance is measured at 593 nm against the blank.

This method has been extensively applied to determine the antioxidant activity of anthocyanins in different matrices: in elderberry [36]; in Roselle extract [37]; in raspberries, blackberries, red currants, gooseberries, and Cornelian cherries [38]; or in carrots, cabbage, cauliflower, potatoes, onions, asparagus and eggplant [39]. However, the results of this method are in the most of the cases compared to the results of other antioxidant assays [40]. Rapid and reproducible results are obtained with this method. However, some limitations related to FRAP assay should be considered. On the one hand, the samples must be aqueous and the pH value is critical. On the other hand, some compounds without antioxidant properties can reduce Fe^3+^ to Fe^2+^ inducing an overestimation of the antioxidant activity of the sample.

### 2.6. CUPRAC (Cupric Ion Reducing Antioxidant Capacity) Assay

CUPRAC assay is a spectrophotometric method similar to FRAP. In this method the anthocyanins react with the CUPRAC reagent (cupric neocuproine) producing the Cu(I)-neocuproine which is a chromophore that absorbs at 450 nm. This method has been successfully applied to various food extracts [7].

The main advantages of this method is related to the positive characteristics of the CUPRAC reagent: availability and easy accessibility, rapidity, stability, low-cost, sensitivity towards thiol-type antioxidants unlike FRAP, and responsiveness to both hydrophilic and lipophilic antioxidants. Nevertheless, the detection is based on the absorption at 450 nm which is non-specific enough and the presence of other compounds present in the sample could be interfered in the results.

### 2.7. ABTS (2,2′-Azino-bis (3-ehtylbenzothiazoline-6-sulfonic acid) Diamonium Salt) Assay

The most recent ABTS assay method is based on decolorization techniques. It consists of the production of a stable radical, blue/green ABTS chromophore, by the reaction of ABTS with potassium persulfate. This stable radical has a maximum absorbance at 415 nm. A drop in absorbance of this compound occurs when the radical reacts with the antioxidant. This method can determine the antioxidant activity of mixtures of substances, helping to distinguish between additive and synergistic effects. The antioxidant activity is calculated relatively to the reactivity of Trolox standard under similar conditions.

This assay is frequently combined with DPPH assay for the determination of antioxidant activity of anthocyanins [41,42,43]. This method is simple and not a large sample volume and time of analysis are necessary. Nevertheless, a standard solution is required in order to obtain accurate results.

This assay is also described in the literature as TEAC [6-hydroxy-2,5,7,8-tetramethylchroman-2-carboxylic acid (Trolox)] equivalent antioxidant capacity. The same free radical is used both TEAC and ABTS assays. However, different reagents have been proposed for the generation of the green–blue ABTS^•+^, resulting in different TEAC assays. This radical is produced by the oxidation of 2,2′-azinobis (3-ethylbenzothiazoline-6-sulfonic acid; ABTS). The oxidation can be reached in different ways: (i) in TEAC assay I, metmyoglobin reacted with H_2_O_2_ generating the ferrylmyoglobin radical, which then reacted with ABTS. (ii) In TEAC assay II, the ABTS^•+^ is formed by the reaction with manganese dioxide. (iii) In TEAC assay III, enzymatic reaction using horseradish peroxidase is applied. Other proposals consist of applying electrochemical oxidation or using 2,2′-azobis-2-amidinopropane, dihydrochloride (AAPH) or potassium persulfate (K_2_S_2_O_8_) as oxidants.

These alternatives are interchangeable when the appropriate solvent is selected. However, differences in assay conditions can be found, such as the reaction time or the wavelength used for the detection, for instance, sometimes in order to avoid interferences 734 nm is preferable to 415 nm.

These assays have been used to determine the antioxidant capacity of the anthocyanins, as a consequence some results has been reported in wine [44]; in corn [45]; in pomegranate juice [46] or in blueberries [47].

The advantages of TEAC assay I are the simplicity, reproducibility, and flexibility to determine the antioxidant capacity in hydrophilic and lipophilic foods. However, the pre-addiction of the sample before the radical generation could result in an overestimation of the antioxidant capacity. Other possible disadvantages of this assay may be the fact that ABTS is not found naturally, and that any compound with a redox potential lower than ABTS^•+^ may react with the radical.

Other alternatives applied to determine the antioxidant activity of anthocyanins involve the use of enzymes [48] or the use of chromatographic techniques. The latter alternative can not only extract the anthocyanins to determine their antioxidant activity [49], but also determining the total anthocyanins concentration (TAC), and the identification and quantification of the presence of each anthocyanin presents in the sample [50,51].

The in vitro assays exposed in this manuscript provide information about the antioxidant activity of the anthocyanins determined by their capacity to neutralize the initiators of the oxidation process (absorbing photons, neutralizing ROS or chelating metals ion) stopping the initiation steps of the autoxidation process. Thus, these assays are based on the reaction of the anthocyanins with: (i) some colored persistent radical such as the free radicals used in DPPH test and TEAC test; or (ii) other oxidizing agents like Fe^3+^ ions used in FRAP test or Cu^2+^ ions used in CUPRAC test. This neutralization capacity is measured under specific conditions of temperature, light, or the combination of both. Other assays to measure the antioxidant capacity of anthocyanins are based on a competitive probe reaction where the competitive reaction of radicals with the anthocyanins or with a probe, is monitored by fluorimetric techniques in the case of the ORAC assay, among others. All of these methods offer information on the actual antioxidant activity. They trap free radical and should be considered as chain-breaking antioxidants. This property can be measured properly only with methods based on inhibited autoxidation, while others (such as DPPH, ABTS) provide only a rough estimation. However, different specific studies have been carried out to determine the preventive antioxidant capacity [52]. Other specific assays to gather information about the inhibition of lipid substrate oxidation, where different factors and mechanism of oxidation are studied simultaneously, can be found in the literature [53].

As it has been explained above, the assays presented in this manuscript follow different mechanisms of reaction to measure the antioxidant capacity. Therefore, the information provided by them and the interpretation of their results should be carefully considered. For example, that is the case of the results provided by FRAP and CUPRAC assays versus ORAC assay which consist in a radical-trapping reaction and directly measures the capacity to neutralize initiators.

Despite the fact that the methods mentioned above are the most commonly used to determine the antioxidant capacity of anthocyanins, there is currently a claim for the development of different alternatives in order to provide direct information about the capacity to prevent the autoxidation in the biological system. Diverse alternatives have been proposed to measure the inhibition of autoxidation. That is the case exposed by Matera et al. [54], where the antioxidant activity is measured by studying the inhibited autoxidation in aqueous micelles. For that purpose, linoleic acid and Triton-X100 were mixed under controlled pH together with an initiator and the extracted anthocyanins. The reaction was tracked by monitoring the oxygen consumption with a Clark-type electrode. Another alternative to measure the capacity of anthocyanins to inhibit autoxidation was proposed by Tisuda et al. [55], where the antioxidative activity was measured by using linoleic acid autoxidation in different systems; liposome, rabbit erythrocyte membrane, and rat liver microsomal. This type of study provides direct information about the relation between the results of antioxidants assays and their biological activity in the prevention of the autoxidation. Currently, the European Food Safety Authority (EFSA) has published a guidance for the scientific requirements for health claims related to antioxidants, oxidative damage, and cardiovascular health. In this guidance, the European Health Claims Regulation has given recommendation to determine the biological effect of food supplements with purported antioxidant activity. According to this document, the antioxidant properties of foods are based on scavenging free radicals that are in vitro studied in model systems and it is not established that this capability has a beneficial physiological effect in humans. Thus, the in vivo human studies in plasma using methods—such as TRAP, TEAC, FRAP, and ORAC—carried out to establish the antioxidant capacity of the plasma do not demonstrate that it has a beneficial physiological effect in humans. Consequently, the protection of cells from premature ageing to promote healthy ageing in relation to the antioxidant properties of food are not sufficiently defined because they are established by non-specific criteria. In order to provide information about the prevention of the autoxidation under specific criteria, the EFSA guidance recommend some assays for specific biological molecules. For example, it proposes: (i) the monitoring of F2-isoprostanes to determine the capacity for the protection of the lipids from oxidative damage; (ii) the analyses of 8-hydroxy-2-deoxy-guanosine in blood, tissue and urine to assess oxidative damage to DNA; and (iii) the monitoring of protein carbonyls by ELISA, that can be applied to determine the protection of protein from oxidation damage [56].

All the assays explained in detail in this manuscript provide results that generally express the antioxidant activity as mmol Trolox Equivalent per kg of fresh weight. However, these results do not report exactly about the antioxidative activity of foods. For this purpose, information about the size portion of each food in the diet is also important to evaluate its antioxidant activity [57]. Due to the relevance of this type of results with regard to the antioxidant activity of anthocyanins, the USDA National Nutrient Database for Standard Reference has published a database where the total concentration of anthocyanins of different fruits and vegetables has been determined together with the service size that should be ingested from each fruit or vegetable [58].

## 3. Classification and Natural Sources of Anthocyanins

Health and therapeutic effects of anthocyanins are related to their chemical and biochemical reactivity, which are partially explained by their antioxidative activities [59,60]. However, the antioxidative activity of anthocyanins does not necessarily transfer to biological activity because any actions on the body depends both on bioavailability and cellular molecular targets [61]. Furthermore, not all the blue, red, and purple fruits, vegetables, and flowers have the same composition and concentration of anthocyanins and in consequence the same antioxidative activity. The fruits with the highest concentration of anthocyanins are berries, currants, grapes, and some tropical fruit. In the group of edible vegetables, leafy vegetables, grains, roots, and tubers show the highest concentration of anthocyanins as well [62]. Furthermore, the presence of anthocyanins can be detected in different parts of the plant such as stem, leaves, and storage organs.

**Figure 1:**
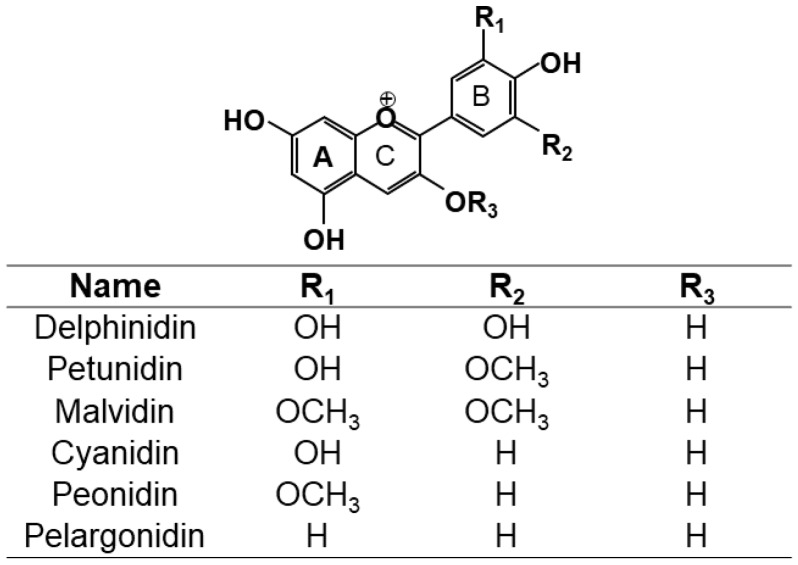
Structure of anthocyanins R_3_ = sugar, and anthocyanidins R_3_ = H.

In nature, these pigments are commonly present as anthocyanin, which is in the form of glycoside, and as anthocyanidin also known as aglycone (one or more saccharide bonded with the aglycone). The base structure of anthocyanins is shown in Figure 1. Nowadays the number of anthocyanins identified in nature is higher than 600 [6]. Among these anthocyanins, glycoside forms of delphinidin, cyanidin, petunidin, peonidin, malvidin, and pelargonidin, are the most abundant [54,55,62,63,64,65]. Table 2 shows the most common anthocyanins that have been identified in different fruits, vegetables, and edible flowers and the numerical code that has been assigned to each anthocyanin to refer them in the manuscript.

In supplementary information, Tables S1–S6 show the most common anthocyanins identified in different fruits, vegetables, and flowers, grouped according to their chemical structure: delphinidin and its derivatives (Table S1), cyanidin and its derivatives (Table S2), petunidin (Table S3), peonidin (Table S4), malvidin (Table S5) and pelargonidin and its derivatives (Table S6). Each table also indicates the natural source of the anthocyanins, the type of extraction that was applied, the chromatographic method used to identify and quantify them and the antioxidant assays applied to determine the antioxidant capacity.

The main conclusion from Table S1 is that delphinidin 3-glucoside (code 4) is the most common and abundant delphinidin in fruits and edible flowers. However, this group of anthocyanins is not so frequent in vegetables, being delphinine the most common. The results showed in Table S2 pointed out that the most common cyanindin in fruit and edible flower is cyanidin 3-glucoside (code 11), being also abundant cyanidin 3-galactoside (code 10) in fruits. In vegetables, cyanidin (code 8) is the most common and abundant but cyanidin 3-glucoside (code 11) is abundant in some grains as well. Table S3 shows that the following anthocyanins can be identified in fruits: petunidin 3-arabinoside, petunidin 3-galactoside, and petunidin 3-glucoside (code 30, 31, 32) in almost all the studied fruits, with petunidin 3-glucoside (code 32) being the most abundant and the only petunidin identified in the flowers. Petunidin (code 29) is the only anthocyanin of this group identified in the studied vegetables. Table S4 reveals that no peonidin was identified in flowers. In fruits, peonidin 3-glucoside (code 37) was the most abundant but also peonidin 3-galactoside (code 36) was identified in the most of them. In vegetables, the most common anthocyanin of this group was peonidin (code 35). From the group of malvidin (Table S5) it can be concluded that the most abundant in fruits is malvidin 3-galactoside (code 51) followed by malvidin 3-glucoside (code 52). The last stage was also the most abundant in flowers followed by malvidin 3,5-diglucoside (code 53). In vegetables, the malvidin was the only anthocyanin quantified of this group. Table S6 shows that pelargonidin 3-glucoside (code 55) was the most abundant in fruits followed by pelargonidin 3-rutinoside (code 56). The pelargonidin 3,5-diglucoside (code 57) was the most abundant in flowers and the pelargonidin (code 54) was the only anthocyanin of this group identified in the studied vegetables.

The protection of these pigments against oxidation process depends on their structures. Not all of them possess the same activities to scavenge diverse reactive oxygen or nitrogen species. The antioxidant ability of anthocyanins depends on the ring orientation since it will determine the willingness to donate a proton and the capacity to transfer and electron. The number of free hydroxyls around the pyrone ring and their positions also play a key role in the antioxidant activity [2]. The presence of other types of radicals in the main structure has an important role in the antioxidant activity as well. Hence, anthocyanins chalcones and quinoidal bases with a double bond conjugated to the keto group are efficient antioxidants at scavenging free radicals. Also, the glycosylated B-ring structure of anthocyanins contributes to the high antioxidant activity, where orthohydroxylation and methoxylation substantially increase the antioxidant activity. Furthermore, anthocyanidins have higher antioxidant activity in comparison with anthocyanins, which has been reported in the literature. The reason may be the lower stability of the anthocyanidin compared to the anthocyanin due to its structure, what consequently makes anthocyanidin highly reactive [66]. Acylation of anthocyanin with one or more phenolic acids has a significant increase in antioxidant activity [54,67], but glycosylation leads to a reduction in the activity [66,68].

**Figure 2:**
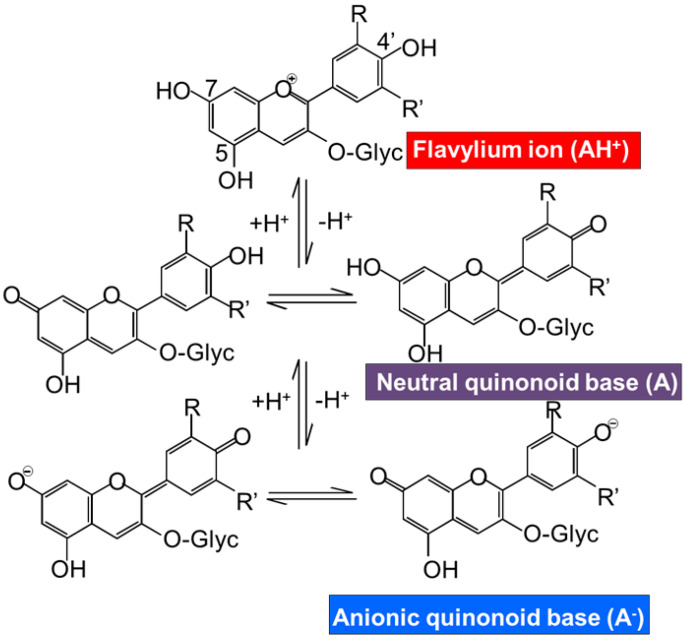
Effect of pH on the structure and color of anthocyanins.

The efficacy of scavenging diverse free radicals differs from one anthocyanin to the other. Pelargonidin-3-glucoside, cyanidin-3-glucoside, and delphinidin-3-glucoside and their standard aglycones have strong antioxidative activity in a liposomal system and reduced formation of malondialdehyde by UVB irradiation [55,69]. Furthermore, the results pointed out the highest inhibitory effect on lipid peroxidation and O_2_^•^ scavenging activity of delphinidin and delphinidin-3-glucoside followed by cyanidin and pelargonidin [2]. On the contrary, pelargonidin had the highest inhibitory effect on hydroxyl radical scavenging activity [66]. Moreover, a study demonstrates the highest inhibitory effect on copper (II)-induced low-density lipoprotein (LDL) oxidation of cyanidin and cyanidin-3-glucoside compared with other phenolic acids, anthocyanins, and anthocyanin aglycones, whereas delphinidin has intermediate efficacy [70]. Another study analyzed the antioxidant activity of malvidin-3-glucoside and the result showed that the quinoidal-base and pseudo-base of malvidin-3-glucoside significantly inhibited peroxidation of linoleate compared with catechin, malvidin, and resveratrol [71]. However, the oxidation activity assigned to these anthocyanins is dependent on the type of reactive species and in consequence on the type of antioxidant assay carried out for the determination of the antioxidant activity. Thus, FRAP and TEAC assays have reported the significant reduction of the antioxidant activity by the metoxilation in the position 5 or 3 and 5 in petunidin and malvidin monoglucoside respectively [72]. Another factor implicated in the reactivity of the anthocyanins and also in their antioxidative activity is the pH. In the literature different studies have demonstrated the effect of the pH in the antioxidant capacity of the anthocyanins from different sources; Roselle [26], wine [71,72], black rice complexed with cycloamylose [73,74], palm juice [75], and *Hibiscus acetosella* [76]. The pH is an important factor that should be controlled in order to determine the reactivity of the anthocyanins. The acid nature of the anthocyanin structure is shown in Figure 2. This acid nature is due to the conjugation of the double bonds in the rings of the main structure and the hydroxyl groups at C4′, C5, and C7 respectively. The hydroxyl group at C7 is the strongest acid. The deprotonation can be produced at acid pH~4 yielding a neutral quinonoid base stabilized by tautomerization with the hydroxyl group at C5. The hydroxyl group at C4 is also susceptible to be deprotonated at higher pH~7 yielding the anionic base. If the pH level is still rising to basic pH, higher than 8, the deprotonation is produced in the C5 yielding the dianionic base which can lead to the chalcone anion [1]. Hence, the pH of the solution controls the proportions of protonated and deprotonated hydrated and isomeric form of anthocyanin, affecting its reactivity. Therefore, the pH should be controlled during the extractions process of anthocyanins and also during the antioxidative bioassays because their results are pH dependent [2,26].

Once the identification of the most common anthocyanins in different foods has been carried out and the influence of the structure and the environment in the capacity of anthocyanins to prevent the oxidation has been discussed, the next step was to determine the bioavailability of these anthocyanins and their implication, enhancing human health.

## 4. Bioavailability of Anthocyanins

The daily intake of anthocyanins can be estimated via food databases and can range from few to hundreds of milligrams per person due to the methodological differences in the assessment, together with the influence of nutritional, cultural, and social differences of the investigated populations [8]. The pattern followed by anthocyanins after oral dispensation is unique and different from other flavonoids [77]. Anthocyanins have a markedly low bioavailability, only 1–2% of the ingested anthocyanins maintain their parent C6–C3–C6 structure in the organism. Food digestion is a pH-dependent process and, therefore, anthocyanins are subjected to transformations in addition to hydrolyzation by several enzymes in the small intestine [12,78]. A portion of the ingested anthocyanins reaches the large intestine, where they are metabolized into low-molecular-weight catabolites, which can be excreted in the feces within 2–4 h (up to 8 h) or absorbed again. Active transporters through either gastric or intestinal cell barrier play an important role in their transfer and absorption within the liver, kidney, brain, or other organs and tissues, besides the stomach [13,79]. In a recent review on tissue bioavailability in animals, Sandoval-Ramírez et al. [80] concluded that the TAC absorbed was 2.17 × 10^5^ pmol/g in mice kidney, 1.73 × 10^5^ pmol/g in liver, 3.6 × 10^3^ pmol/g in heart, and 1.16 × 10^5^ pmol/g in lung; and 6.08 × 10^3^ pmol/g in pig brain. In the wall of the intestine and then in the liver, anthocyanins and their catabolites undergo phase 2 enzymatic metabolism being also transformed into their glucuronidated, sulphated, and methylated forms [10,12,13,14,78,81,82,83]. The presence of microbial catabolites at many sites of the body, at higher concentration than the native form, has suggested that part of the biological activities attributed to anthocyanins is related to the synergetic effect of their colonic catabolites [13,84]. Anthocyanin metabolites and transformation products have been characterized and quantified by several authors [85,86,87,88,89]. Ferrars et al. [88] identified a wide variety of anthocyanin phenolic metabolites, including 11 novel metabolites, in post-menopausal women after 12 weeks elderberry intake, at concentration levels higher than their anthocyanin native forms. There are many critical factors affecting the fate of anthocyanins and their metabolites in our organism: the ability to cross membranes, pH, digestive enzymes, microbiota, biliary acids, or food matrix. The use of radiolabeled (^14^C) or stable isotope–labelled (^13^C) tracer studies provides useful information about in which extent anthocyanins are metabolized to phenolic acid derivatives. In this sense, Czank et al. [90] investigated the fate of anthocyanins in eight male participants after the ingestion of ^13^C-cyanidin-3-*O*-glucoside (500 mg). The relative mean bioavailability was 12.38% (5.37% excreted in urine and 6.91% in breath). The authors found maximum serum concentration 42-fold higher for ^13^C-labeled metabolites than their respective native compound ^13^C-cyanidin-3-glucoside. Up to 49 metabolites were detected including among others: phase II conjugates of cyanidin-3-glucoside and cyanidin (cyanidin-glucuronide, methyl cyanidin-glucuronide, and methyl cyanidin-3-glucoside-glucuronide); degradation products (protocatechuic acid, phloroglucinaldehyde, and phloroglucinaldehyde); phase II conjugates of protocatechuic acid, phenylacetic acids, phenylpropenoic acids, and hippuric acid.

The mechanisms through which anthocyanins may exert their bioactivity are not fully understood as it is not clear whether their activity is linked to native forms, their derivatives, or both. The distinction of their different biological roles is a very challenging task. Some comparative studies have been conducted on the antioxidant activity of anthocyanin metabolites [91]. Recently, Kim et al. [92] provided basic information of the chemical changes of cyanidin glycosides during in vitro gastrointestinal digestion. Cyanidin-3-*O*-galactoside was degraded into caffeoylquinic acid, which was not found after in vitro digestion of cyanidin-3-*O*-glucoside. The bioactivity (DPPH) of the anthocyanin metabolites decreased in the intestinal fraction. However, the bioactivity increased after simulated colonic digestion, possibly because of the newly formed colonic metabolites. Furthermore, anthocyanin metabolites from the chokeberry extract exhibited higher DPPH radical activities than those from the mulberry extract. In another study, α-glucosidase inhibitory activity and ROS scavenging activities of conjugated-pelargonidin-3-*O*-glucoside samples were potentially increased after gastrointestinal digestion [93].

**Figure 3:**
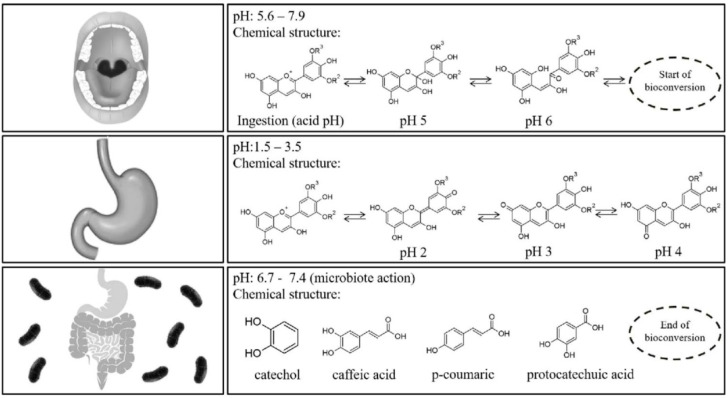
Schematic representation of the anthocyanins chemical structures influenced by the digestion process steps (R_2_ and R_3_ = H or Methyl) (taken from Braga et al. [14], with permission of Elsevier).

A scheme on the physicochemical reactions observed during the three main stages of the human digestion process can be observed in Figure 3 [11]. Biotransformation reactions start in the oral cavity through salivary amylase (pH 5.6–7.9). Once in the stomach at pH 1.5–3.5, anthocyanins exist in multiple ionic forms being mainly present as red flavylium cations and quinoidal blue species. Finally, in the intestinal step (pH 6.7–7.4) anthocyanins are present as colorless carbinol (with limited absorption) and occur the biotransformation into low molecular weight molecules such as phenolic acids or catechol (gallic acid, vanillic acid, protocatechuic acid, 4-hydroxybenzoic acid, and syringic acid have been identified as the main degradation products of delphinidin-3-*O*-glucoside, peonidin-3-*O*-glucoside, cyanidin-3-*O*-glucoside, pelargonidin-3-*O*-glucoside and malvidin-3-*O*-glucoside, respectively) [13].

**Figure 4:**
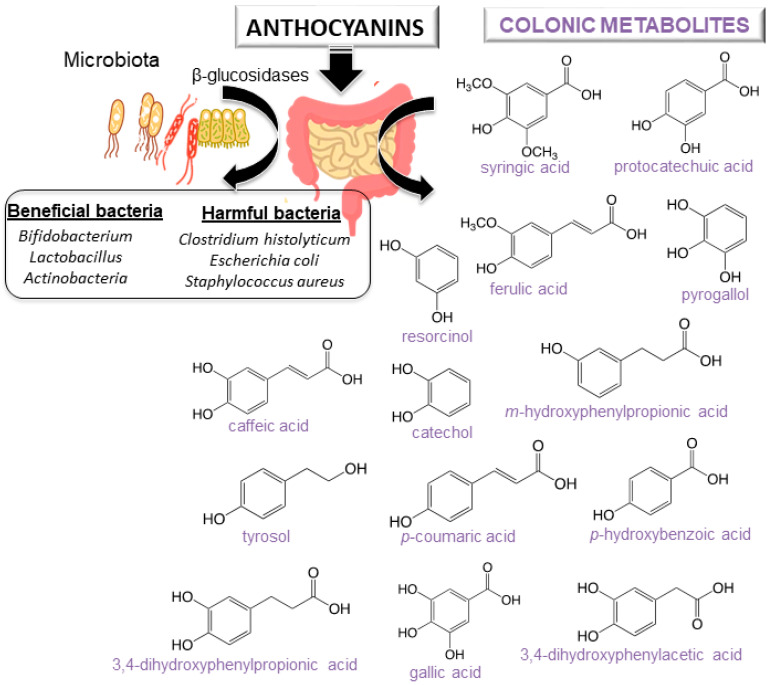
Scheme of anthocyanins metabolites from colonic metabolism and colon microbiota alteration.

For a better understanding of the anthocyanin bioavailability, different in vivo and in vitro models simulating digestion have been proposed [11]. Gowd et al. [94] assessed the phenolic profile of blackberry anthocyanin extract followed by human gut microbiota fermentation at different time intervals (0–48 h). Authors revealed the formation of gut metabolites enhance the high glucose plus palmitic acid induced ROS, mitochondrial membrane collapse, and glutathione depletion in HepG2 cells. Several studies have also reported that after anthocyanin colonic fermentation occurs an increase of beneficial bacteria (*Bifidobacterium* spp., *Actinobacteria*, *Bacteroidetes*, *Lactobacillus/Enterococcus* spp., *Akkermansia*) [95,96,97,98,99,100]. Intestinal microbiota possesses β-glucosidase activity, allowing the release of glucose from the aglycone and providing energy to support bacterial growth. A study recently carried out by Zhou et al. [95] suggests that the consumption of blueberry and its extracts could exert prebiotic activity and a modulatory effect on the composition and abundance of human intestinal microbiota. Anthocyanins could enhance human health by modulating gut microorganisms, which are often related to different diseases [95,101]. Nevertheless, it is important to note that anthocyanin derivatives can also reduce some harmful bacteria such as *C. histolyticum* after colonic fermentation [101,102]. A summary of anthocyanins colonic metabolism metabolites and colon microbiota alteration is shown in Figure 4 [11].

In any case, there are still many doubts on mechanisms involved and which factors have crucial impact on bioavailability [7]. Anthocyanins with efficient effect for one individual may not have the same effect for another [12] and there is still a high variability in the results obtained. Based on the literature and recent reviews [11,14] this variability is due to the lack of homogeneity introduced at three levels: (i) food matrix and food processing; (ii) enzymatic levels (affected by genetic factors and diet, age, and sex); and (iii) microbiota functionality. Reported data considering inter- or intra-individual variability is very scarce and bioavailability methods are not standardized making very difficult to reach firm conclusions. On the one hand, in vitro methods (cell-based assays) fail to consider the role of the individual microbiota present in the human body; while, on the other hand, in in vivo trials (human trials and animal studies) each subject has their own microbiota [14].

It is also important to note that the incorporation of anthocyanins into food and medical products is a challenging task due to their high instability and susceptibility to degradation. In this sense, the use of nano/microencapsulation with natural polymers is one of the best strategies to improve the stability of sensitive substances in in vitro simulated gastrointestinal digestion and colonic fermentation [103,104]. According to a recent review on this topic [104] different techniques have been tested to encapsulate anthocyanins including spray-drying > freeze-drying > gelation > lipid-based particles > electrohydrodynamic processes. The first one is the most economical, simplest, and the most applied method (80–90%) [105,106]. The use of other techniques still remains poorly explored probably due to the hydrophilic nature of anthocyanins, being therefore a promising area of future research [104,107,108,109].

Blackberry anthocyanins encapsulated with β-cyclodextrin [101,110] or gum arabic [111] helped to delay the release of anthocyanins during in vitro simulated gastrointestinal digestion. The stability of anthocyanins can be also influenced by the type of wall material. Recently, Wu et al. [103] evaluated the effect of four different wall materials during in vitro simulated digestion and colonic fermentation. The encapsulation technique enhanced significantly the colonic accessibility and delayed the release of anthocyanins, especially for soy protein. Degradation products of anthocyanins such as syringic acid produced during colonic fermentation by the action of gut microbiota were indicative of their benefits for host health.

## 5. Therapeutic Effects of Anthocyanins

Available scientific studies prove the beneficial effects of the presence of anthocyanins in fruits and vegetables in the prevention of diseases [60,66,81,112]. Even after the ingestion of high doses of anthocyanin and derivatives no negative effects have been observed [113]. This section covers the main health benefits of anthocyanins in different types of pathologies including eye health, cardiovascular disease, antiobesity, antidiabetic, antimicrobial effects, anticancer activities, and neurodegenerative disorders. A summary of the positive effects of anthocyanins is shown in Table 3 [114,115,116,117,118,119,120,121,122,123,124,125,126,127,128,129,130,131,132,133,134,135,136,137,138,139,140,141,142,143,144,145,146,147,148,149,150,151,152,153,154,155,156,157,158,159,160], and their mechanisms of action in disease prevention are discussed below.

Eye health: Since the first report in 1966 about the positive effects of anthocyanins on vision in humans, anthocyanin-rich extracts have been worldwide utilized as a popular supplement for ocular health [161,162]. Oral dispensation of blackcurrant anthocyanins may be a promising supplement for patients with open-angle glaucoma, being also effective for antiglaucoma medication, while anthocyanin-rich bilberry extract has a protective effect on vision during retinal inflammation [115]. It has also been confirmed that cyanidin helps the regeneration of rhodopsin and smooth muscle relaxation in rats [116]. Results have also showed that bilberry extracts were able to suppress the photoxidation of pyridinium disretinoid A2E, an auto-fluorescence pigment that accumulates in retinal epithelial cells with age and can cause light-induced damage to the cell. In a comparative study a significant improvement on nocturnal visual function and an improved contrast sensitivity levels in subjects with myopia versus placebo group was observed [114]. Anthocyanins act also inhibiting transient myopia, reducing eye fatigue or enhancing retinal blood flow with glaucoma [118,121,161,163].

Cardiovascular diseases: It is especially important the role of anthocyanins in preventing myocardial infarction and cardiovascular disease related to mortality. Extracts of anthocyanins have been used to inhibit platelet aggregation being preventive in the initial stage of thrombi; in the treatment of problem with poor micro-circulation resulting from capillary fragility; and also to prevent the LDL oxidation [122,164,165,166]. In a placebo-controlled trial in dyslipidemia patients (40–65 years) the intake of berry-derived anthocyanins improved lipoprotein profile through cholesteryl ester transfer protein inhibition [123]. Authors observed a greater increase in high-density lipoprotein (HDL) cholesterol levels and in the cellular cholesterol efflux to serum as well as a decrease in LDL cholesterol levels in the anthocyanin group in contrast to the placebo group. Similar results were reported by Álvarez Suárez et al. [127] in an in vivo study using healthy volunteers supplemented with strawberries (500 g). Daily consumption improved the lipid profile reducing total cholesterol, LDL cholesterol and triglycerides levels, while HDL cholesterol remained unchanged. This increased antihemolytic defenses and platelet function in the subjects. In another attempt, higher intakes of fruit-based anthocyanins were associated to a lower risk of nonfatal myocardial infarction (14%) and ischemic stroke in a prospective cohort study in men over 24 years [124]. A meta-analysis of 45 randomized controlled trials stated that the consumption of berries and purified anthocyanins (2.2−1230 mg anthocyanins/day) increases significantly HDL-cholesterol and reduces LDL-cholesterol, triglycerides, systolic blood pressure, and diastolic blood pressure as well as the inflammatory markers CRP and TNFα [167]. The analysis also suggested that some individuals are more susceptible to the protective effects of anthocyanin consumption: (i) overweight; (ii) over 50 years; and (iii) those with increased risk of cardiovascular disease. Another meta-analysis of 99 randomized controlled trials showed that the consumption of anthocyanin rich-products decreased significantly both systolic and diastolic blood pressure regardless of the health status of the participants [168].

In in vitro assays, anthocyanins have also shown inhibition of the porcine pancreatic elastase [169], an enzyme that plays a significant function in pathologies such as arteriosclerosis, emphysema, or rheumatoid arthritis, etc., by attacking fibers and collagen. Moreover, acceleration in the cicatrization process due to anthocyanin-rich extract has been demonstrated, showing preventive and curative activity against gastroduodenal ulcers induced in rats [7]. Their influence on the biosynthesis of mucopolysaccharides provably improves the efficacy of the gastric mucous layer, and increases the base substance of the connective tissue and of the capillaries [170].

Antiobesity and Antidiabetic effects: Anthocyanins have shown anti-obesity effects through multiple mechanisms such as inhibiting lipid absorption, regulating lipid metabolism, increasing energy expenditure, suppressing food intake and regulating gut microbiota, which suggests anthocyanins are promising candidates in anti-obesity therapies [171]. Kwon et al. [128] observed that anthocyanins-added diet from black soybean in rats decreases body weight gains, being significantly lowered in the rats fed with a high fat diet plus black soybean anthocyanins compared with the rats fed with high fat diet without black soybean. Anthocyanins also improved the lipid profile and suppressed the high fat diet-induced weight gain in liver intermediately and decreased the weights of epididymal and perirenal fat pads.

In addition, type 2 diabetes is closely related to obesity [66]. Anthocyanins can alleviate complications in type 2 diabetes by inhibiting intestinal glucose absorption, inducing pancreatic insulin secretion, upregulating glucose transporter type 4, and suppressing hepatic gluconeogenesis [172]. After the supplementation of a high-fat diet during 13 weeks with different berries in mice, Heyman et al. [173] observed that those supplemented mice gained lesser body weight and presented lower fasting insulin levels than the control group as well as mediated positive effects on glucose homeostasis. Jankowski et al. [130] described a substantial decrease in the sugar concentration in urine and blood serum after streptozotocin injection in fed rats with grapes. The mechanisms of anthocyanins suggested by the authors were the reduction of the biosynthesis of collagen, lipoproteins, and glycoproteins, as well as the reduction of the activity of elastase and adenosine deaminase (both high in diabetic patients). Treatment with cherries in rats resulted in a significant reduction of blood glucose and urinary microalbumin and an increase of the creatinine secretion level in urea [174]. The pulp, seed and skin from “red chilto” (a red fruit from Argentina) had a hypoglycemic effect and acted increasing glucose absorption, decreasing glucose diffusion rate and promoting glucose transport across the cell membrane [175] in an in vitro simulated gastroduodenal digestion. Consumption of blueberries and apples/pears in humans was also associated to a lower risk of type 2 diabetes [176].

Antimicrobial effects: The antimicrobial activity of anthocyanins against a wide range of microorganisms is also well documented. Possible mechanisms induced cell damage by destroying the cell wall, membrane and intercellular matrix [66,140,177]. Blackberry extracts have antibacterial activity with the highest sensitivity to *Aeromonas hydrophilia* and *Listeria innocua* [141]. Cranberry extracts have antibacterial activity towards *Enterococcus faecium* resistant to vancomycin, *Pseudomonas aeruginosa*, *Staphylococcus aureus*, and *Escherichia coli* [142]. Different types of berry extracts inhibit Gram-negative bacteria but not Gram-positive bacteria [143] probably because Gram-negative bacteria acts as a preventive barrier against hydrophobic compounds but not against hydrophilic compounds [178].

Anticancer activity: Possible mechanisms of the anticancer activity of anthocyanins have been described by many authors: antimutagenic activity; inhibition of oxidative DNA damage and carcinogen activation; induction of phase II enzymes for detoxification; cell cycle arrest; inhibition of cyclooxygenase-2 enzymes; as well as induction of apoptosis and antiangiogenesis [179,180,181,182,183,184].

In breast cancer, anthocyanins cause the inhibition of key modulators that promote its progression and development by acting directly in the DNA fragmentation and promoting the death of MCF-7 cancer cells [185,186]. In addition, the studies indicate that anthocyanins exert extensive in vitro anti-invasive and in vivo anti-metastatic activities. For example, delphinidin can act as a potential antimetastatic agent that suppresses PMA-induced cancer cell invasion through the specific inhibition of NF-κB-dependent MMP-9 gene expression [187,188]. In lung cancer, the treatment of cyanidin-3-glucoside and cyanidin 3-rutinoside, isolated from mulberry, inhibits the migration and invasion of A549 cells and also decreases MMP-2 and uPA and enhances TIMP-2 and PAI. Anthocyanins also inhibit the growth of carcinogenic cells that provoke colon cancer, induce the apoptosis effect, and are even able to act as modulators of the macrophages in the immune response [180]. Forester et al. [189] also reported the positive effect of anthocyanin metabolites decreasing cell viability and causing cell cycle arrest and apoptosis in colon cancer. In oral and cervical cancer, the invasion of SCC-4 cells and HeLa cells were diminished by the treatment of peonidin 3-glucoside and cyanidin-3-glucoside [190].

It is also important to note that the structures of anthocyanins have a considerable influence on their biological activities [191,192,193]. In this sense, the type of aglycones, sugars, and acylated acids, and the position and degree of glycosylation and acylation seem to be the main factors influencing the anticancer property [191]. Jing et al. [192] compared the anticancer properties of anthocyanin-rich extracts using human colon cancer HT29 cell line. Authors reported the following growth inhibitory activity rates: purple corn > chokeberry and bilberry > purple carrot and grape > radish and elderberry. Those non-acylated monoglycosylated anthocyanins had greater anticancer property than those with pelargonidin, triglycoside, and/or acylation with cinnamic acid.

Neurodegenerative diseases: Anthocyanins are also uniquely suited for the treatment of neurodegenerative diseases such as Alzheimer’s, Parkinson’s, or amyotrophic lateral sclerosis. Their main mechanisms include antioxidant pathways, calcium homeostasis, inflammation, protein homeostasis, and the balance of pro-survival and pro-apoptotic signaling [194,195].

In a primary cell model of Parkinson’s disease, dopaminergic cell death elicited by rotenone was suppressed by extracts prepared from blueberries, grape seed, hibiscus, blackcurrant, and mulberry [154]. Moreover, Strathearn et al. [154] observed that those extracts rich in anthocyanins and proanthocyanidins exhibited greater neuroprotective activity than extracts rich in other polyphenols.

The oral dispensation of anthocyanins (200 mg/kg) in rats was able to regulate cholinergic neurotransmission, to restore Na^+^, K^+^-ATPase and Ca^2+^-ATPase activities, and to prevent memory deficits caused by scopolamine dispensation [156]. Rehman et al. [157] showed the neuroprotective effect of anthocyanins based on an artificial ageing model using D-galactose to induce oxidative stress and inflammatory response. The potential mechanisms of their action included: decreased expression of the receptor for advance glycation end product, reduced level of ROS, and lipid peroxidation. Shih et al. [155] observed that mice fed with anthocyanin-rich mulberry extracts demonstrated significantly less amyloid β protein and showed improvement of learning and memory ability in avoidance response tests. The fed mice also showed a higher antioxidant enzyme activity and less lipid oxidation in both brain and liver, as compared to the control mice. Besides, the treatment with anthocyanin-rich mulberry extract has been proved to decrease the levels of serum aspartate aminotransferase, alanine aminotransferase, triglyceride, and total cholesterol that increase with ageing.

Furthermore, the therapeutic profile of anthocyanins can be improved by encapsulation [158,159,160]. For instance, in Alzheimer’s disease Amin et al. [158] showed that encapsulated nanoparticles loaded with anthocyanins are rapidly taken up by cells enhancing their neuroprotective profile against amyloid beta toxicity above that of anthocyanins alone. Similar activity was also observed in in vivo studies in mice [158,160].

## 6. Conclusions

In order to establish the antioxidant activity of anthocyanins and how their intake affects the human health, many factors should be taken into account. Firstly, an evaluation of the antioxidant activity from a multiparametric perspective is requires as the total concentration, the structure, the nature of the sample, the pH and the mechanism of the reaction play an important role in their effect. On a regular basis, more than one antioxidant assay—one for each mechanism—should be carried out. Besides identification and quantification of the anthocyanins is also highly recommended to establish an accurate value for the antioxidant activity of a sample. Secondly, it is required to know the bioavailability of these compounds after their intake. Many studies that have demonstrated the benefits of anthocyanin-rich extracts in the prevention of diseases. Nonetheless, it is important to note that their efficacy depends on their bioavailability. Along the digestion process, anthocyanins are metabolized into various conjugates, which then ultimately metabolize into phenolic acid degradation products as well. The accumulated evidence suggests the synergy effect between all possible forms to explain their attributed health-promoting properties. An inter- and intra-individual variability in anthocyanins absorption, metabolism, distribution, and excretion is also evident. Among the main factors that probably affect this variability are: food matrix and processing, enzymatic levels and microbiota functionality. Attention should be paid in different lines: (i) to perform well-designed standardized methods when evaluating bioavailability; (ii) to consider inter or intra-individual variability in anthocyanin metabolism; (iii) to assess the antioxidant activity of anthocyanin metabolites during gastrointestinal digestion; and, since anthocyanin might exert different biological activities, (iv) to draw a more accurate characterization profiles.

## Figures and Tables

**Table 1:** Commonly used methods for measurement in vitro of antioxidant activity

Bioassay	Reagents Involved in the Reaction	Detection	Method
DPPH (Diphenyl-1-picrylhydrazyl) assay	Free radical (DPPH^•+^)	Decrease of Abs. at 515 nm	Spectrophotometric or colorimetric
ORAC Assay (Oxygen Radical Absorbance Capacity)	2,2′-azobis(2-amidino-propane) dihydrochloride (AAPH) to produce free radical β-phycoerythrin or Fluorescein or Pyrogallol red	Decrease of fluorescence	Fluorescence spectroscopy
TRAP assay (total peroxyl radical trapping antioxidant parameter)	2,2′-azobis(2-amidopropane) hydrochloride (ABAP) to produce free radical Luminol	Decrease of luminescence	Chemiluminescence
FCT (ferric thiocyanate) assay	Ferrous chloride, formation of red ferric thiocyanate	Increase of Abs. at 500 nm	Spectrophotometric
FRAP (Ferric Reducing Antioxidant Power) assay	FeCl_3_·6H_2_O, formation of blue ferrous complexes	Increase of Abs. at 593 nm	Colorimetric
CUPRAC, Cupric Ion Reducing Antioxidant Capacity	Cupric neocuproine, formation of Cu(I)-neocuproine	Increase of Abs. at 550 nm	Spectrophotometric
ABTS [2,2′-azino-bis (3-ehtylbenzothiazoline-6-sulfonic acid) diamonium salt] assay	Free radical (ABTS^•+^)	Decrease of Abs. at 415 nm	Colorimetric
Methods of inhibited autoxidation	Lipid molecules, azoinitiator	O_2_ consumption/hydroperoxide formation	Oxygen electrode, pressure gauge, detection of conjugated dienes

Note: Abs, absorbance.

**Table 2:** Anthocyanins identified in different fruits, vegetables, and edible flowers and their codes.

Code	Anthocyanin	Code	Anthocyanin
1	Delphinidin	30	Petunidin 3-arabinoside
2	Delphinidin 3-arabinoside	31	Petunidin 3-galactoside
3	Delphinidin 3-galactoside	32	Petunidin 3-glucoside
4	Delphinidin 3-glucoside	33	Petunidin 3-halactoside
5	Delphinidin 3,5-diglucoside	34	Petunidin 3-rutinoside
6	Delphinidin 3-rutinoside	35	Peonidin
7	Delphinidin 3-*O*-(6″-*p*-coumaroyl-glucoside)	36	Peonidin 3-galactoside
8	Cyanidin	37	Peonidin 3-glucoside
9	Cyanidin 3-arabidoside	38	Peonidin 3-rutinoside
10	Cyanidin 3-galactoside	39	Peonidin 3-(6′-malonylglucoside)
11	Cyanidin 3-glucoside	40	Peonidin 3-(3″,6″-dimalonylglucoside)
12	Cyanidin 3,5-diglucoside	41	Peonidin 3-glucoside/malvidin 3-galactoside
13	Cyanidin 3-rutinoside	42	Peonidin 3-arabinoside/malvidin 3-glucoside
14	Cyanidin 3-(6′-malonylglucoside)	43	Peonidin 3-*O*-sophoroside-5-*O*-glucoside
15	Cyanidin 3-(3″,6″-dimalonylglucoside)	44	Peonidin 3-*p*-hydroxybenzoylsophoroside-5-glucoside
16	Cyanidin 3-xyloside	45	Peonidin 3-caffeoylsophoroside-5-glucoside
17	Cyanidin 3-xylosylrutinoside	46	Peonidin 3-dicaffeoylsophoroside-5-glucoside
18	Cyanidin 3-dioxaloylglucoside	47	Peonidin 3-caffeoyl-*p*-hydroxybenzoylsophoroside-5-glucoside
19	Cyanidin 3-halavtoside	48	Peonidin 3-caffeoy-feruloylsophoroside-5-glucoside
20	Cyanidin 3-*O*-sophoroside	49	Malvidin
21	Cyanidin 3-sophoroside-5-rhamnoside	50	Malvidin 3-arabinoside
22	Cyanidin 3-sambubioside	51	Malvidin 3-galactoside
23	Cyanidin 3-sambubioside-5-rhamnoside	52	Malvidin 3-glucoside
24	Cyanidin-3-*p*-hydroxybenzoylsophoroside-5-glucoside	53	Malvidin 3,5-diglucoside
25	Cyanidin-3-caffeoylsophoroside-5-glucoside	54	Pelargonidin
26	Cyanidin-3-caffeoyl-*p*-hydroxybenzoylsophoroside-5-glucoside	55	Pelargonidin 3-glucoside
27	Cyanidin 3-(*p*-coumaroyl)-diglucoside-5-glucoside	56	Pelargonidin 3-rutinoside
28	Cyanidin 3-(*p*-coumaroyl)-diglucoside-5-glucoside	57	Pelargonidin 3,5-diglucoside
29	Petunidin		

**Table 3:** Health benefits of anthocyanins.

Eye Health	Administration	References
Improvement of vision in patients with open-angle glaucoma	Oral capsule	[114]
Protective effect during retinal inflammation	IV in rats	[115]
Regeneration of rhodopsin and smooth muscle relaxation	IV in mouse model	[116]
Improvement of dark adaptation	Oral capsule	[117]
Prevention of cataractogenesis of diabetic cataract	Incubation of Enucleated rat lenses	[118]
Antiapoptotic effects against oxidative damage of lens epithelial cell	Cell studies	[119]
Prevention of retinal degeneration induced by N-methyl-N-nitrosourea	Oral solution	[120]
Increase of ocular blood flows	Oral capsule	[121]
**Cardiovascular diseases**		
Inhibition of platelet aggregation (in vitro antithrombotic properties)	Cell studies	[122]
Increase of high-density lipoprotein cholesterol levels and decrease of low-density lipoprotein cholesterol levels	Oral capsule	[123]
Lower risk of non-fatal myocardial infarction	Oral intake	[124]
Vasorelaxation properties in isolated coronary artery rings in pigs	Cell studies	[125]
Decrease of susceptibility to ischemia-reperfusion injury and infarct size	Rodent food	[126]
Improvement of lipid profile and platelet function	Oral capsule	[127]
**Antiobesity effects**		
Improvement of weight gain and lipid profile on obese rats	Fat diet-induced mouse model	[128]
Suppression of body weight gain and improve blood lipid profile in rats	Fat diet-induced mouse model	[129]
Reduction of sugar concentration in urine and plasma in rats	Intraperitoneal and intragastric administration	[130]
Ameliorated obesity in high-fat-fed mice	Cell studies	[131]
Upregulation of adipocytokine secretion and gene expression in rat adipocytes	Cell studies	[132]
Suppression of fat tissue gain, weight gain and other metabolic disorders	Fat diet-induced mouse model	[133]
**Antidiabetic effects**		
Amelioration of hyperglycemia and insulin sensitivity in diabetic mice	Fat diet-induced mouse model	[134]
Improvement of dyslipidemia, enhancement of antioxidant capacity, and prevention of insulin resistance in human with type 2 diabetes	Oral capsule	[135]
Alleviation of glomerular angiogenesis of diabetic kidneys in mice	Cell studies	[136]
Inhibition of DPP IV activity (a protease that regulates blood glucose levels via degradation of incretins)	Computational studies	[137]
Amelioration of renal apoptosis in diabetic nephropathy mice	Oral solution	[138]
Activation of adipose tissue-derived adiponectin to defend against diabetes-related endothelial dysfunction in mice	Diet-induced mouse model	[139]
**Antimicrobial effects**		
Induction of cell damage by destroying the cell wall, membrane, and intercellular matrix	Cell studies	[140]
Highest sensitivity to *Aeromonas hydrophila* and *Listeria innocua*	Microbial strains	[141]
Antibacterial effects towards *Enterococcus faecium* resistant to vancomycin, *Pseudomonas aeruginosa*, *Staphylococcus aureus* and *Escherichia coli*	Microbial strains	[142]
Inhibition of Gram-negative bacteria	Microbial strains	[143]
**Anticancer effects**		
Suppression of cell proliferation, inflammation, and angiogenesis and induction of apoptosis in esophageal tissue of rats	Diet-induced rat model	[144]
Anti-invasive potential in breast cancer cell lines	Cell studies	[145]
Anticancer effect on BALB/c nude mice bearing MDA-MB-453 cell xenografts and breast cancer cell lines	Cell studies	[146]
Inhibition of cell migration and invasion, suppression of activation of rapidly accelerated fibrosarcoma, mitogen-activated protein kinase and c-Jun N-terminal kinase, and downregulation of secretion of matrix metalloproteinase 2	Cell studies	[147]
Inhibition of growth of human HT-29 colon cancer cells, increase of expression of tumor suppression genes and decrease of cyclooxygenase-2 gene expression	Cell studies	[148]
Reduction of colonic aberrant crypt foci, colonic cellular proliferation and COX-2 mRNA expression in rats	Diet-induced rat model	[149]
Suppression of formation of aberrant crypt foci in colons of CF-1 mice	Cell studies and diet-induced rat model	[150]
Promotion of apoptosis in benign prostatic hyperplasia rats	Oral doses in rat model	[151]
Anti-invasive effect on human hepatoma Hep3B cells and inhibition of matrix metalloproteinase MMP-2 and MMP-9 gene expression	Cell studies	[152]
Inhibition of Akt-mTOR signaling thereby inducing maturation of acute myeloid leukemia cells, besides inducing apoptotic players such as TRAIL in cancer systems	Cell studies	[153]
**Neurodegenerative diseases**		
Neuroprotective activity by suppression of dopaminergic cell death in Parkinson’s disease	Cell studies	[154]
Improvement of learning and memory ability in mice. Higher antioxidant enzyme activity and less lipid oxidation in both brain and liver	Diet-induced mouse model	[155]
Regulation of cholinergic neurotransmission to restore Na+, K^+^-ATPase and Ca^2+^-ATPase activities and to prevent memory deficits in rats	Oral and injected rat models	[156]
Neuroprotective effect: Memory and synaptic dysfunction	Oral rat models	[157]
Improvement of its free radical scavenging capabilities via p38/JNK pathway against Abeta1-42-induced oxidative stress	Cell studies	[158]
Enhancement of neuroprotection against Abeta1-42-induced neuroinflammation and neurodegeneration	Oral mouse model and cell studies	[159]
Enhancement of the neuroprotection in an Abeta1-42 mouse model of Alzheimer’s disease	Oral mouse model and cell studies	[160]

## Supplementary Materials

The following are available online at https://www.mdpi.com/2076-3921/9/5/451/s1, Table S1: Natural source, type of extraction, chromatographic method and antioxidant assay applied to identify and quantify delphinidin and its derivatives identified with codes, Table S2: Natural source, type of extraction, chromatographic method and antioxidant assay applied to identify and quantify cyanidin and its derivatives identified with codes, Table S3: Natural source, type of extraction, chromatographic method and antioxidant assay applied to identify and quantify petunidin and its derivatives identified with codes, Table S4: Natural source, type of extraction, chromatographic method and antioxidant assay applied to identify and quantify peonidin and its derivatives identified with codes, Table S5: Natural source, type of extraction, chromatographic method and antioxidant assay applied to identify and quantify malvidin and its derivatives identified with codes, Table S6: Natural source, type of extraction, chromatographic method and antioxidant assay applied to identify and quantify pelargonidin and its derivatives identified with codes.
