# Supplementary material for: State of the Art of Anthocyanins: Antioxidant Activity, Sources, Bioavailability, and Therapeutic Effect in Human Health
Source: Antioxidants (Basel). 2020 May 23;9(5):451. doi: 10.3390/antiox9050451 (PMC7278599; doi:10.3390/antiox9050451)
Supplement: Supplementary file 1 [file antioxidants-09-00451-s001.pdf]

SUPPLEMENTARY MATERIAL

State of the Art of Anthocyanins: Antioxidant Activity, Sources, Bioavailability and Therapeutic Effect in Human Health

Noelia Tena [1,*], Julia Martín [2], Agustin G. Asuero [1]

[1] Departamento de Química Analítica, Facultad de Farmacia, Universidad de Sevilla, Prof. García González, 2, 41012, Sevilla, España; noelia.tena@us.es (N.T.); asuero@us.es (A.G.A.).
[2] Departamento de Química Analítica, Escuela Politécnica Superior, Universidad de Sevilla, C/ Virgen de África 7, E-41011 Sevilla, España; jbueno@us.es (J.M.)
* Correspondence: ntena@us.es

Table S1. Natural source, type of extraction, chromatographic method and antioxidant assay applied to identify and quantify delphinidin and its derivatives identified with codes.

	Delphinidin and its derivatives							Extraction, identification, quantification and antioxidant activity	References
**Code**	**1**	**2**	**3**	**4**	**5**	**6**	**7**		
Raspberry (mg/g)	-	-	-	-	-	-	-	Freeze dried fruit, 80% MeOH 0.5% AcOH. HPLC-ESI-MS/MS; HPLC-DAD. DPPH, ABTS, FRAP.	[63]
Bilberry (mg/g)	-	3.5	4.6	4.7	-	-	-		
Blackberry (mg/g)	-	-	-	-	-	-	-		
Blueberry (mg/g)	-	0.7	1.4	0.1	-	-	-		
Blueberry Wild (mg/100 g FW)	-	57.4 - 85.2	65.3 - 124.7	78.9 - 133.9	-	-	-	Ground fruit (85:15 v/v, MeOH:HCl) RP-HPLC-DAD. DPPH, ABTS, FRAP,ORAC.	[47]
Blueberry monocultivar (mg/100 g FW)	-	12.5 - 46.2	ND - 60.3	21.7 - 60.6	-	-	-		
Cranberry (mg/g)	-	-	-	-	-	-	-	Freeze dried fruit, 80% MeOH 0.5% AcOH. HPLC-ESI-MS/MS; HPLC-DAD. DPPH, ABTS, FRAP.	[63]
Crowberry (mg/g)	-	1.4	8.6	0.4	-	-	-		
Mulberry (mg/g)	-	-	-	-.	-	-	-		
Blackcurrant (mg/g)	-	-	-	2.9	-	9.8	-		
Blackcurrant Commercial (%)	-	-	-	11.8 - 15.1	-	31.7 - 35.7	-	Fresh berries extracted with 95% (v/v) ethanol acidified with 0.1N HCl. HPLC–UV–MS	[50]
Redcurrant (mg/g)	-	-	-	-	-	-	-	Freeze dried fruit, 80% MeOH 0.5% AcOH. HPLC-ESI-MS/MS; HPLC-DAD. DPPH, ABTS, FRAP.	[63]
Strawberry (mg/g)	-	-	-	-	-	-	-		
Pomegranate juice (mg/L)	-	-	-	138.2 - 147.4	85.5 - 93.0	-	-	Juice clarified 5 mL 15% gelatine HPLC, TEAC	[46]
Green rye grains (mg/Kg DW)	-	-	-	ND - <LOQ	-	<LOQ - 0.2	-	Dried and ground grain 70% EtOH and 1% (w/v) citric acid, purification, HPLC-ESI-MS	[64]
Violet rye grains (mg/Kg DW)	-	-	-	-	-	-	-		
Red Chicory (mg/100 g FW)	-	-	-	-	-	-	-	Ground sample MeOH and 0.03% HCl) HPLC-PDA/-ESI+-MS. ABTS, CRUPAC, DPPH	[42]
Red Onion (mg/100 g FW)	-	-	-	-	-	-	8.7		
Red onion (mg/100 g DW)	ND	-	-	-	-	-	-	Freeze powder sample MeOH 0.1% HCl, UPLC, LC–DAD–ESI–MS. DPPH, FRAP, ORAC	[39]
Eggplant (mg/100 g FW)	-	-	-	-	-	-	-	Ground sample MeOH and 0.03% HCl) HPLC-PDA/-ESI+-MS. ABTS, CRUPAC, DPPH	[42]
Eggplant Black Beauty (mg/100 g DW)	11.5 ± 0.8	-	-	-	-	-	-	Freeze powder sample MeOH 0.1% HCl, UPLC, LC–DAD–ESI–MS. DPPH, FRAP, ORAC	[39]
Purple sweet potato (mg/100 g FW)	-	-	-	-	-	-	-	Ground sample MeOH and 0.03% HCl) HPLC-PDA/-ESI+-MS. ABTS, CRUPAC, DPPH	[42]

Purple potato (mg/100 g DW)	0.3 - 0.6	-	-	-	-	-	-	Freeze powder sample MeOH 0.1% HCl, UPLC, LC–DAD–ESI–MS. DPPH, FRAP, ORAC	[39]
Red potato (mg/100 g DW)	ND	-	-	-	-	-	-		
Black carrot (mg/100 g FW)	-	-	-	-	-	-	-	Ground sample MeOH and 0.03% HCl) HPLC-PDA/-ESI+-MS. ABTS, CRUPAC, DPPH	[42]
Purple carrot (mg/100 g DW)	ND - 0.3	-	-	-	-	-	-	Freeze powder sample MeOH 0.1% HCl, UPLC, LC–DAD–ESI–MS. DPPH, FRAP, ORAC	[39]
Red cabbage Gario (mg/100 g DW)	ND	-	-	-	-	-	-		
Purple cauliflower Graffitti (mg/100 g DW)	ND	-	-	-	-	-	-		
Purple asparagus Albenga (mg/100 g DW)	ND	-	-	-	-	-	-		
Begonia, light red (µg/g)	-	-	-	ND	-	-	-	Maceration of flower (1:10) (80% MeOH, 19% H_2O and 1% HCl, v/v/v). HPLC-DAD DPPH, FRAP, ORAC	[65]
Torenia, purple (µg/g)	-	-	-	210.9- 211.0	-	-	-		
Mini rose, dark red (µg/g)	-	-	-	ND	-	-	-		
Clitoria, blue (µg/g)	-	-	-	ND	-	-	-		
Mini daisy, white (µg/g)	-	-	-	11.1 - 11.2	-	-	-		
Tagete, orange (µg/g)	-	-	-	ND	-	-	-		
Cosmos, yellow (µg/g)	-	-	-	0.3	-	-	-		
Cravine, red wine (µg/g)	-	-	-	ND	-	-	-		

Note: ND, not detected; 1, Delphinidin; 2, Delphinidin 3-arabinoside; 3, Delphinidin 3-galactoside; 4, Delphinidin 3-glucoside; 5, Delphinidin 3,5-diglucoside; 6 Delphinidin 3-rutinoside; 7, Delphinidin 3-O-(6”-p-coumaroyl-glucoside)

Table S2. Natural source, type of extraction, chromatographic method and antioxidant assay applied to identify and quantify cyanidin and its derivatives identified with codes. Continue….

	Cyanidin and its derivatives part 1							Extraction, identification, quantification and antioxidant activity	References
**Code**	**8**	**9**	**10**	**11**	**12**	**13**	**14**		
Raspberry (mg/g)	-	-	-	1	-	0.3	-	Freeze dried fruit, 80% MeOH 0.5% AcOH. HPLC-ESI-MS/MS; HPLC-DAD. DPPH, ABTS, FRAP.	[63]
Bilberry (mg/g)	-	2.5	3.7	4.1	-		-		
Blackberry (mg/g)	-	0.1	-	7.2	-	0.1	-		
Blueberry (mg/g)	-	0.1	0.3	0.1	-	-	-		
Blueberry Wild (mg/100 g FW)	-	-	33.7 - 101.8	37.2 - 107.5	-	-	-	Ground fruit (85:15 v/v, MeOH:HCl) RP-HPLC-DAD. DPPH, ABTS, FRAP,ORAC.	[47]
Blueberry monocultivar (mg/100 g FW)	-	-	ND - 11.8	0.1 - 3.7	-	-	-		
Cranberry (mg/g)	-	0.6	3.9	0.3	-	-	-	Freeze dried fruit, 80% MeOH 0.5% AcOH. HPLC-ESI-MS/MS; HPLC-DAD. DPPH, ABTS, FRAP.	[63]
Crowberry (mg/g)	-	2.3	8	0.6	-	-	-		
Mulberry (mg/g)	-	-	-	9.6	-	4.2	-		
Blackcurrant (mg/g)	-	-	-	1.1	-	7.1	-		
Blackcurrant Commercial (%)	-	-	-	6.8 - 10.6		41.1-47.1	-	Fresh berries extracted with 95% (v/v) ethanol acidified with 0.1N HCl. HPLC–UV–MS	[50]
Redcurrant (mg/g)	-	-	-	-	-	0.4	-	Freeze dried fruit, 80% MeOH 0.5% AcOH. HPLC-ESI-MS/MS; HPLC-DAD. DPPH, ABTS, FRAP.	[63]
Strawberry (mg/g)	-	-	-	0.1	-	-	-		
Pomegranate juice (mg/L)	-	-	-	219.2 - 239.2	168.0 - 178.8	-	-	Juice clarified 5 mL 15% gelatine HPLC, TEAC	[46]
Green rye grains (mg/Kg DW)	-	-	-	-	-	<LOQ - 0.1	-	Dried and ground grain 70% EtOH and 1% (w/v) citric acid, purification, HPLC-ESI-MS	[64]
Violet rye grains (mg/Kg DW)	-	-	-	ND - 40.1	-	ND - 0.7	-		
Red Chicory (mg/100 g FW)	-	-.	-	30.9	-	-	-	Ground sample MeOH and 0.03% HCl) HPLC-PDA/-ESI+-MS. ABTS, CRUPAC, DPPH	[42]
Red Onion (mg/100 g FW)	-	-	-	4.4	-	-	-		
Red onion (mg/100 g DW)	3.0 - 5.9	-	-	-	-	-	-	Freeze powder sample MeOH 0.1% HCl, UPLC, LC–DAD–ESI–MS. DPPH, FRAP, ORAC	[39]
Eggplant (mg/100 g FW)	-	-	-	-	-	-	-	Ground sample MeOH and 0.03% HCl) HPLC-PDA/-ESI+-MS. ABTS, CRUPAC, DPPH	[42]

Eggplant Black Beauty (mg/100 g DW)	ND	-	-	-	-	-	-	Freeze powder sample MeOH 0.1% HCl, UPLC, LC–DAD–ESI–MS. DPPH, FRAP, ORAC	[39]
Purple sweet potato (mg/100 g FW)	-	-	-	-	-	-	-	Ground sample MeOH and 0.03% HCl) HPLC-PDA/-ESI+-MS. ABTS, CRUPAC, DPPH	[42]
Purple potato (mg/100 g DW)	0.1 - 0.2	-	-	-	-	-	-	Freeze powder sample MeOH 0.1% HCl, UPLC, LC–DAD–ESI–MS. DPPH, FRAP, ORAC	[39]
Red potato (mg/100 g DW)	0.5 - 0.6	-	-	-	-	-	-		
Black carrot (mg/100 g FW)	-	-	-	-	-	-	-	Ground sample MeOH and 0.03% HCl) HPLC-PDA/-ESI+-MS. ABTS, CRUPAC, DPPH	[42]
Purple carrot (mg/100 g DW)	13.1 - 18.6	-	-	-	-	-	-	Freeze powder sample MeOH 0.1% HCl, UPLC, LC–DAD–ESI–MS. DPPH, FRAP, ORAC	[39]
Red cabbage Gario (mg/100 g DW)	52.1 ± 64.1	-	-	-	-	-	-		
Purple cauliflower Graffitti (mg/100 g DW)	61.5 - 65.9	-	-	-	-	-	-		
Purple asparagus Albenga (mg/100 g DW)	8.0 -8.2	-	-	-	-	-	-		
Begonia, light red (µg/g)	-	-	-	ND	ND	-	-	Maceration of flower (1:10) (80% MeOH, 19% H_2O and 1% HCl, v/v/v). HPLC-DAD DPPH, FRAP, ORAC	[65]
Torenia, purple (µg/g)	-	-	-	ND	ND	-	-		
Mini rose, dark red (µg/g)	-	-	-	ND	ND	-	-		
Clitoria, blue (µg/g)	-	-	-	4.7 -4.8	3.2 -3.3	-	-		
Mini daisy, white (µg/g)	-	-	-	ND	ND	-	-		
Tagete, orange (µg/g)	-	-	-	ND	ND	-	-		
Cosmos, yellow (µg/g)	-	-	-	ND	ND	-	-		
Cravine, red wine (µg/g)	-	-	-	17.3- 17.5	ND	-	-		

Note: ND, not detected; 8, Cyanidin; 9, Cyanidin 3-arabidoside; 10, Cyanidin 3-galactoside; 11, Cyanidin 3-glucoside; 12, Cyanidin 3,5-diglucoside; 13, Cyanidin 3-rutinoside; 14, Cyanidin 3-(6'-malonylglucoside).

Table S2. Natural source, type of extraction, chromatographic method and antioxidant assay applied to identify and quantify cyanidin and its derivatives identified with codes.

	Cyanidin and its derivatives part 2														Extraction, identification, quantification and antioxidant activity	References
**Code**	**15**	**16**	**17**	**18**	**19**	**20**	**21**	**22**	**23**	**24**	**25**	**26**	**27**	**28**		
Raspberry (mg/g)	-	-	-	-	-	6.3	1.2	-	0.5	-	-	-	-	-	Freeze dried fruit, 80% MeOH 0.5% AcOH. HPLC-ESI-MS/MS; HPLC-DAD. DPPH, ABTS, FRAP.	[63]
Bilberry (mg/g)	-	-	-	-	-	-	-	-	-	-	-	-	-	-		
Blackberry (mg/g)	-	0.5	-	2.1	-	-	-	-	-	-	-	-	-	-		
Blueberry (mg/g)	-	-	-	-	-	-	-	-	-	-	-	-	-	-		
Blueberry Wild (mg/100 g FW)	-	-	-	-	-	-	-	-	-	-	-	-	-	-	Ground fruit (85:15 v/v, MeOH:HCl) RP-HPLC-DAD. DPPH, ABTS, FRAP,ORAC.	[47]
Blueberry monocultivar (mg/100 g FW)	-	-	-	-	-	-	-	-	-	-	-	-	-	-		
Cranberry (mg/g)	-	-	-	-	-	-	-	-	-	-	-	-	-	-	Freeze dried fruit, 80% MeOH 0.5% AcOH. HPLC-ESI-MS/MS; HPLC-DAD. DPPH, ABTS, FRAP.	[63]
Crowberry (mg/g)	-	-	-	-	-	-	-	-	-	-	-	-	-	-		
Mulberry (mg/g)	-	-	-	-	0.1	-	-	-	-	-	-	-	-	-		
Blackcurrant (mg/g)	-	-	-	-	-	-	-	-	-	-	-	-	-	-		
Blackcurrant Commercial (%)	-	-	-	-	-	-	-	-	-	-	-	-	-	-	Fresh berries extracted with 95% (v/v) ethanol acidified with 0.1N HCl. HPLC–UV–MS	[50]
Redcurrant (mg/g)	-	-	1.8	-	-	-	-	0.3	-	-	-	-	-	-	Freeze dried fruit, 80% MeOH 0.5% AcOH. HPLC-ESI-MS/MS; HPLC-DAD. DPPH, ABTS, FRAP.	[63]
Strawberry (mg/g)	-	-	-	-	-	-	-	-	-	-	-	-	-	-		
Pomegranate juice (mg/L)	-	-	-	-	-	-	-	-	-	-	-	-	-	-	Juice clarified 5 mL 15% gelatine HPLC, TEAC	[46]
Green rye grains (mg/Kg DW)	-	-	-	-	-	-	-	-	-	-	-	-	-	-	Dried and ground grain 70% EtOH and 1% (w/v) citric acid, purification, HPLC-ESI-MS	[64]
Violet rye grains (mg/Kg DW)	ND - 9.1	-	-	-	-	-	-	-	-	-	-	-	-	-		
Red Chicory (mg/100 g FW)	-	-	-	-	-	-	-	-	-	-	-	-	-	-	Ground sample MeOH and 0.03% HCl) HPLC-PDA/-ESI+-MS. ABTS, CRUPAC, DPPH	[42]
Red Onion (mg/100 g FW)	-	-	-	-	-	-	-	-	-	-	-	-	-	-		
Red onion (mg/100 g DW)	-	-	-	-	-	-	-	-	-	-	-	-	-	-	Freeze powder sample MeOH 0.1% HCl, UPLC, LC–DAD–ESI–MS. DPPH, FRAP, ORAC	[39]

Eggplant (mg/100 g FW)	-	-	-	-	-	-	-	-	-	-	-	-	-	-	Ground sample MeOH and 0.03% HCl) HPLC-PDA/-ESI+-MS. ABTS, CRUPAC, DPPH	[42]
Eggplant Black Beauty (mg/100 g DW)	-	-	-	-	-	-	-	-	-	-	-	-	-	-	Freeze powder sample MeOH 0.1% HCl, UPLC, LC–DAD–ESI–MS. DPPH, FRAP, ORAC	[39]
Purple sweet potato (mg/100 g FW)	-	-	-	-	-	-	-	-	-	1.0	0.4	2.4	-	-	Ground sample MeOH and 0.03% HCl) HPLC-PDA/-ESI+-MS. ABTS, CRUPAC, DPPH	[42]
Purple potato (mg/100 g DW)	-	-	-	-	-	-	-	-	-	-	-	-	-	-	Freeze powder sample MeOH 0.1% HCl, UPLC, LC–DAD–ESI–MS. DPPH, FRAP, ORAC	[39]
Red potato (mg/100 g DW)	-	-	-	-	-	-	-	-	-	-	-	-	-	-		
Black carrot (mg/100 g FW)	-	-	-	-	-	-	-	-	-	-	-	-	21.2	0.7	Ground sample MeOH and 0.03% HCl) HPLC-PDA/-ESI+-MS. ABTS, CRUPAC, DPPH	[42]
Purple carrot (mg/100 g DW)	-	-	-	-	-	-	-	-	-	-	-	-	-	-	Freeze powder sample MeOH 0.1% HCl, UPLC, LC–DAD–ESI–MS. DPPH, FRAP, ORAC	[39]
Red cabbage Gario (mg/100 g DW)	-	-	-	-	-	-	-	-	-	-	-	-	-	-		
Purple cauliflower Graffitti (mg/100 g DW)	-	-	-	-	-	-	-	-	-	-	-	-	-	-		
Purple asparagus Albenga (mg/100 g DW)	-	-	-	-	-	-	-	-	-	-	-	-	-	-		
Begonia, light red (µg/g)	-	-	-	-	-	-	-	-	-	-	-	-	-	-	Maceration of flower (1:10) (80% MeOH, 19% H_2O and 1% HCl, v/v/v). HPLC-DAD DPPH, FRAP, ORAC	[65]
Torenia, purple (µg/g)	-	-	-	-	-	-	-	-	-	-	-	-	-	-		
Mini rose, dark red (µg/g)	-	-	-	-	-	-	-	-	-	-	-	-	-	-		
Clitoria, blue (µg/g)	-	-	-	-	-	-	-	-	-	-	-	-	-	-		
Mini daisy, white (µg/g)	-	-	-	-	-	-	-	-	-	-	-	-	-	-		
Tagete, orange (µg/g)	-	-	-	-	-	-	-	-	-	-	-	-	-	-		
Cosmos, yellow (µg/g)	-	-	-	-	-	-	-	-	-	-	-	-	-	-		
Cravine, red wine (µg/g)	-	-	-	-	-	-	-	-	-	-	-	-	-	-		

Note: ND, not detected; 15, Cyanidin 3-(3'',6''-dimalonylglucoside); 16, Cyanidin 3-xyloside; 17, Cyanidin 3-xylosylrutinoside; 18, Cyanidin 3-dioxaloylglucoside; 19, Cyanidin 3-halavtoside; 20, Cyanidin 3-O-sophoroside; 21, Cyanidin 3-sophoroside-5-rhamnoside; 22, Cyanidin 3-sambubioside; 23, Cyanidin 3-sambubioside-5-rhamnoside; 24, Cyanidin-3-p-hydroxybenzoylsophoroside-5-glucoside; 25, Cyanidin-3-caffeoylsophoroside-5-glucoside; 26, Cyanidin-3-caffeoyl-p-hydroxybenzoylsophoroside-5-glucoside; 27, Cyanidin 3-(p-coumaroyl)-diglucoside-5-glucoside; 28, Cyanidin 3-(p-coumaroyl)-diglucoside-5-glucoside.

Table S3. Natural source, type of extraction, chromatographic method and antioxidant assay applied to identify and quantify petunidin and its derivatives identified with codes.

Code	Petunidin and its derivatives						Extraction, identification, quantification and antioxidant activity	References
	29	30	31	32	33	34		
Raspberry (mg/g)	-	-	-	-	-	-	Freeze dried fruit, 80% MeOH 0.5% AcOH. HPLC-ESI-MS/MS; HPLC-DAD. DPPH, ABTS, FRAP.	[63]
Bilberry (mg/g)	-	0.8	-	2.9	1.5	-		
Blackberry (mg/g)	-	-	-	-	-	-		
Blueberry (mg/g)		0.5	1.1	0.1				
Blueberry Wild (mg/100 g FW)	-	8.4 - 14.7	16.6 - 33.2	77.4 - 165.3	-	-	Ground fruit (85:15 v/v, MeOH:HCl) RP-HPLC-DAD. DPPH, ABTS, FRAP,ORAC.	[47]
Blueberry monocultivar (mg/100 g FW)	-	ND - 14.6	ND - 26.0	7.3 - 27.6	-	-		
Cranberry (mg/g)	-	-	-	-	-	-	Freeze dried fruit, 80% MeOH 0.5% AcOH. HPLC-ESI-MS/MS; HPLC-DAD. DPPH, ABTS, FRAP.	[63]
Crowberry (mg/g)	-	0.5	3.8	0.2	-	-		
Mulberry (mg/g)	-	-	-	-	-	-		
Blackcurrant (mg/g)	-	-	-	-	-	0.18		
Blackcurrant Commercial (%)	-	-	-	-	-	-	Fresh berries extracted with 95% (v/v) ethanol acidified with 0.1N HCl. HPLC–UV–MS	[50]
Redcurrant (mg/g)	-	-	-	-	-	-	Freeze dried fruit, 80% MeOH 0.5% AcOH. HPLC-ESI-MS/MS; HPLC-DAD. DPPH, ABTS, FRAP.	[63]
Strawberry (mg/g)	-	-	-	-	-	-		
Pomegranate juice (mg/L)	-	-	-	-	-	-	Juice clarified 5 mL 15% gelatine HPLC, TEAC	[46]
Green rye grains (mg/Kg DW)	-	-	-	-	-	-	Dried and ground grain 70% EtOH and 1% (w/v) citric acid, purification, HPLC-ESI-MS	[64]
Violet rye grains (mg/Kg DW)	-	-	-	-	-	-		

Red Chicory (mg/100 g FW)	-	-	-	-	-	-	Ground sample MeOH and 0.03% HCl) HPLC-PDA/-ESI+-MS. ABTS, CRUPAC, DPPH	[42]
Red Onion (mg/100 g FW)	-	-	-	-	-	-		
Red onion (mg/100 g DW)	ND	-	-	-	-	-	Freeze powder sample MeOH 0.1% HCl, UPLC, LC–DAD–ESI–MS. DPPH, FRAP, ORAC	[39]
Eggplant (mg/100 g FW)	-	-	-	-	-	-	Ground sample MeOH and 0.03% HCl) HPLC-PDA/-ESI+-MS. ABTS, CRUPAC, DPPH	[42]
Eggplant Black Beauty (mg/100 g DW)	0.5 - 0.6	-	-	-	-	-	Freeze powder sample MeOH 0.1% HCl, UPLC, LC–DAD–ESI–MS. DPPH, FRAP, ORAC	[39]
Purple sweet potato (mg/100 g FW)	-	-	-	-	-	-	Ground sample MeOH and 0.03% HCl) HPLC-PDA/-ESI+-MS. ABTS, CRUPAC, DPPH	[42]
Purple potato (mg/100 g DW)	13.1 - 31.0	-	-	-	-	-	Freeze powder sample MeOH 0.1% HCl, UPLC, LC–DAD–ESI–MS. DPPH, FRAP, ORAC	[39]
Red potato (mg/100 g DW)	-	-	-	-	-	-		
Black carrot (mg/100 g FW)	-	-	-	-	-	-	Ground sample MeOH and 0.03% HCl) HPLC-PDA/-ESI+-MS. ABTS, CRUPAC, DPPH	[42]
Purple carrot (mg/100 g DW)	ND - 0.3	-	-	-	-	-	Freeze powder sample MeOH 0.1% HCl, UPLC, LC–DAD–ESI–MS. DPPH, FRAP, ORAC	[39]
Red cabbage Gario (mg/100 g DW)	ND	-	-	-	-	-		
Purple cauliflower Graffitti (mg/100 g DW)	ND	-	-	-	-	-		
Purple asparagus Albenga (mg/100 g DW)	ND	-	-	-	-	-		
Begonia, light red (µg/g)	-	-	-	ND	-	-	Maceration of flower (1:10) (80% MeOH, 19% H_2O and 1% HCl, v/v/v). HPLC-DAD DPPH, FRAP, ORAC	[65]
Torenia, purple (µg/g)	-	-	-	ND	-	-		
Mini rose, dark red (µg/g)	-	-	-	ND	-	-		
Clitoria, blue (µg/g)	-	-	-	27.5 - 27-6	-	-		
Mini daisy, white (µg/g)	-	-	-	ND	-	-		
Tagete, orange (µg/g)	-	-	-	ND	-	-		
Cosmos, yellow (µg/g)	-	-	-	ND	-	-		
Cravine, red wine (µg/g)	-	-	-	ND	-	-		

Note: ND, not detected; 29, Petunidin; 30, Petunidin 3-arabinoside; 31, Petunidin 3-galactoside; 32, Petunidin 3-glucoside; 33, Petunidin 3-halactoside; 34, Petunidin 3-rutinoside.

Table S4. Natural source, type of extraction, chromatographic method and antioxidant assay applied to identify and quantify peonidin and its derivatives identified with codes. Continue….

Code	Peonidin and derivatives part 1							Extraction, identification, quantification and antioxidant activity	References
	35	36	37	38	39	40	41		
Raspberry (mg/g)	-	-	-	-	-	-	-	Freeze dried fruit, 80% MeOH 0.5% AcOH. HPLC-ESI-MS/MS; HPLC-DAD. DPPH, ABTS, FRAP.	[63]
Bilberry (mg/g)	-	0.5	-	-	-	-	3.5		
Blackberry (mg/g)	-	-	-	-	-	-	-		
Blueberry (mg/g)	-	0.2	-	-	-	-	3.7		
Blueberry Wild (mg/100 g FW)	-	ND - 10.0	26.9 - 120.8	-	-	-	-	Ground fruit (85:15 v/v, MeOH:HCl) RP-HPLC-DAD. DPPH, ABTS, FRAP,ORAC.	[47]
Blueberry monocultivar (mg/100 g FW)	-	ND - 137.8	11.2 - 61.9	-	-	-	-		
Cranberry (mg/g)	-	-	0.1	-	-	-	-	Freeze dried fruit, 80% MeOH 0.5% AcOH. HPLC-ESI-MS/MS; HPLC-DAD. DPPH, ABTS, FRAP.	[63]
Crowberry (mg/g)	-	-	-	-	-	-	-		
Mulberry (mg/g)	-	-	-	-	-	-	-		
Blackcurrant (mg/g)	-	-	-	-	-	-	-		
Blackcurrant Commercial (%)	-	-	-	-	-	-	-	Fresh berries extracted with 95% (v/v) ethanol acidified with 0.1N HCl. HPLC–UV–MS	[50]
Redcurrant (mg/g)	-	-	-	-	-	-	-	Freeze dried fruit, 80% MeOH 0.5% AcOH. HPLC-ESI-MS/MS; HPLC-DAD. DPPH, ABTS, FRAP.	[63]
Strawberry (mg/g)	-	-	-	-	-	-	-		

Pomegranate juice (mg/L)	-	-	-	-	-	-	-	Juice clarified 5 mL 15% gelatine HPLC, TEAC	[46]
Green rye grains (mg/Kg DW)	-	-	-	-	-	-	-	Dried and ground grain 70% EtOH and 1% (w/v) citric acid, purification, HPLC-ESI-MS	[64]
Violet rye grains (mg/Kg DW)	-	-	0.1- 34.0	ND - 0.7	ND - 26.1	ND - 5.9	-		
Red Chicory (mg/100 g FW)	-	-	-	-	-	-	-	Ground sample MeOH and 0.03% HCl) HPLC-PDA/-ESI+-MS. ABTS, CRUPAC, DPPH	[42]
Red Onion (mg/100 g FW)	-	-	-	-	-	-	-		
Red onion (mg/100 g DW)	ND	-	-	-	-	-	-	Freeze powder sample MeOH 0.1% HCl, UPLC, LC–DAD–ESI–MS. DPPH, FRAP, ORAC	[39]
Eggplant (mg/100 g FW)	-	-	-	-	-	-	-	Ground sample MeOH and 0.03% HCl) HPLC-PDA/-ESI+-MS. ABTS, CRUPAC, DPPH	[42]
Eggplant Black Beauty (mg/100 g DW)	ND	-	-	-	-	-	-	Freeze powder sample MeOH 0.1% HCl, UPLC, LC–DAD–ESI–MS. DPPH, FRAP, ORAC	[39]
Purple sweet potato (mg/100 g FW)	-	-	0.5	-	-	-	-	Ground sample MeOH and 0.03% HCl) HPLC-PDA/-ESI+-MS. ABTS, CRUPAC, DPPH	[42]
Purple potato (mg/100 g DW)	0.3 - 0.9	-	-	-	-	-	-	Freeze powder sample MeOH 0.1% HCl, UPLC, LC–DAD–ESI–MS. DPPH, FRAP, ORAC	[39]
Red potato (mg/100 g DW)	1.3 - 2.5	-	-	-	-	-	-		
Black carrot (mg/100 g FW)	-	-	-	-	-	-	-	Ground sample MeOH and 0.03% HCl) HPLC-PDA/-ESI+-MS. ABTS, CRUPAC, DPPH	[42]
Purple carrot (mg/100 g DW)	0.3 - 0.5	-	-	-	-	-	-	Freeze powder sample MeOH 0.1% HCl, UPLC, LC–DAD–ESI–MS. DPPH, FRAP, ORAC	[39]
Red cabbage Gario (mg/100 g DW)	0.6 - 0.7	-	-	-	-	-	-		
Purple cauliflower Graffitti (mg/100 g DW)	ND	-	-	-	-	-	-		
Purple asparagus Albenga (mg/100 g DW)	ND	-	-	-	-	-	-		
Begonia, light red (µg/g)	-	-	-	-	-	-	-	Maceration of flower (1:10) (80% MeOH, 19% H_2O and 1% HCl, v/v/v). HPLC-DAD DPPH, FRAP, ORAC	[65]
Torenia, purple (µg/g)	-	-	-	-	-	-	-		
Mini rose, dark red (µg/g)	-	-	-	-	-	-	-		
Clitoria, blue (µg/g)	-	-	-	-	-	-	-		
Mini daisy, white (µg/g)	-	-	-	-	-	-	-		
Tagete, orange (µg/g)	-	-	-	-	-	-	-		
Cosmos, yellow (µg/g)	-	-	-	-	-	-	-		
Cravine, red wine (µg/g)	-	-	-	-	-	-	-		

Note: ND, not detected; 35, Peonidin; 36, Peonidin 3-galactoside; 37, Peonidin 3-glucoside; 38 Peonidin 3-rutinoside; 39, Peonidin 3-(6'-malonylglucoside); 40, Peonidin 3-(3",6"-dimalonylglucoside); 41, Peonidin 3-glucoside/malvidin 3-galactoside.

Table S4. Natural source, type of extraction, chromatographic method and antioxidant assay applied to identify and quantify peonidin and its derivatives identified with codes.

	Peonidin and derivatives part 2							Extraction, identification, quantification and antioxidant activity	References
**Code**	**42**	**43**	**44**	**45**	**46**	**47**	**48**		
Raspberry (mg/g)		-	-	-	-	-	-	Freeze dried fruit, 80% MeOH 0.5% AcOH. HPLC-ESI-MS/MS; HPLC-DAD. DPPH, ABTS, FRAP.	[63]
Bilberry (mg/g)	3.6	-	-	-	-	-	-		
Blackberry (mg/g)		-	-	-	-	-	-		
Blueberry (mg/g)	0.4	-	-	-	-	-	-		
Blueberry Wild (mg/100 g FW)	-	-	-	-	-	-	-	Ground fruit (85:15 v/v, MeOH:HCl) RP-HPLC-DAD. DPPH, ABTS, FRAP,ORAC.	[47]
Blueberry monocultivar (mg/100 g FW)	-	-	-	-	-	-	-		
Cranberry (mg/g)	-	-	-	-	-	-	-	Freeze dried fruit, 80% MeOH 0.5% AcOH. HPLC-ESI-MS/MS; HPLC-DAD. DPPH, ABTS, FRAP.	[63]
Crowberry (mg/g)	1.6	-	-	-	-	-	-		
Mulberry (mg/g)	-	-	-	-	-	-	-		
Blackcurrant (mg/g)	-	-	-	-	-	-	-		
Blackcurrant Commercial (%)	-	-	-	-	-	-	-	Fresh berries extracted with 95%	[50]

								(v/v) ethanol acidified with 0.1N HCl. HPLC–UV–MS	
Redcurrant (mg/g)	-	-	-	-	-	-	-	Freeze dried fruit, 80% MeOH 0.5% AcOH. HPLC-ESI-MS/MS; HPLC-DAD. DPPH, ABTS, FRAP.	[63]
Strawberry (mg/g)	-	-	-	-	-	-	-		
Pomegranate juice (mg/L)	-	-	-	-	-	-	-	Juice clarify 5 mL 15% gelatine HPLC, TEAC	[46]
Green rye grains (mg/Kg DW)	-	-	-	-	-	-	-	Dried and ground grain 70% EtOH and 1% (w/v) citric acid, purification, HPLC-ESI-MS	[64]
Violet rye grains (mg/Kg DW)	-	-	-	-	-	-	-		
Red Chicory (mg/100 g FW)	-	-	-	-	-	-	-	Ground sample MeOH and 0.03% HCl) HPLC-PDA/-ESI+-MS. ABTS, CRUPAC, DPPH	[42]
Red Onion (mg/100 g FW)	-	-	-	-	-	-	-		
Red onion (mg/100 g DW)	-	-	-	-	-	-	-	Freeze powder sample MeOH 0.1% HCl, UPLC, LC–DAD–ESI–MS. DPPH, FRAP, ORAC	[39]
Eggplant (mg/100 g FW)	-	-	-	-	-	-	-	Ground sample MeOH and 0.03% HCl) HPLC-PDA/-ESI+-MS. ABTS, CRUPAC, DPPH	[42]
Eggplant Black Beauty (mg/100 g DW)	-	-	-	-	-	-	-	Freeze powder sample MeOH 0.1% HCl, UPLC, LC–DAD–ESI–MS. DPPH, FRAP, ORAC	[39]
Purple sweet potato (mg/100 g FW)	-	0.8	3.4	3.9	10.2	15.2	4.5	Ground sample MeOH and 0.03% HCl) HPLC-PDA/-ESI+-MS. ABTS, CRUPAC, DPPH	[42]
Purple potato (mg/100 g DW)	-	-	-	-	-	-	-	Freeze powder sample MeOH 0.1% HCl, UPLC, LC–DAD–ESI–MS. DPPH, FRAP, ORAC	[39]
Red potato (mg/100 g DW)	-	-	-	-	-	-	-		
Black carrot (mg/100 g FW)	-	-	-	-	-	-	-	Ground sample MeOH and 0.03% HCl) HPLC-PDA/-ESI+-MS. ABTS, CRUPAC, DPPH	[42]
Purple carrot (mg/100 g DW)	-	-	-	-	-	-	-	Freeze powder sample MeOH 0.1% HCl, UPLC, LC–DAD–ESI–MS. DPPH, FRAP, ORAC	[39]
Red cabbage Gario (mg/100 g DW)	-	-	-	-	-	-	-		
Purple cauliflower Graffitti (mg/100 g DW)	-	-	-	-	-	-	-		
Purple asparagus Albenga (mg/100 g DW)	-	-	-	-	-	-	-		
Begonia, light red (µg/g)	-	-	-	-	-	-	-	Maceration of flower (1:10) (80% MeOH, 19% H_2O and 1% HCl, v/v/v). HPLC-DAD DPPH, FRAP, ORAC	[65]
Torenia, purple (µg/g)	-	-	-	-	-	-	-		
Mini rose, dark red (µg/g)	-	-	-	-	-	-	-		
Clitoria, blue (µg/g)	-	-	-	-	-	-	-		
Mini daisy, white (µg/g)	-	-	-	-	-	-	-		
Tagete, orange (µg/g)	-	-	-	-	-	-	-		
Cosmos, yellow (µg/g)	-	-	-	-	-	-	-		
Cravine, red wine (µg/g)	-	-	-	-	-	-	-		

Note: ND, not detected; 42, Peonidin 3-arabinoside/malvidin 3-glucoside; 43, Peonidin 3-O-sophoroside-5-O-glucoside; 44, Peonidin 3-p-hydroxybenzoylsophoroside-5-glucoside; 45, Peonidin 3-caffeoylsophoroside-5-glucoside; 46, Peonidin 3-dicaffeoylsophoroside-5-glucoside; 47, Peonidin 3-caffeoyl-p-hydroxybenzoylsophoroside-5-glucoside; 48, Peonidin 3-caffeoy-feruloylsophoroside-5-glucoside.

Table S5. Natural source, type of extraction, chromatographic method and antioxidant assay applied to identify and quantify malvidin and its derivatives identified with codes.

	Malvidin and its derivatives					Extraction, identification, quantification and antioxidant activity	References
**Code**	**49**	**50**	**51**	**52**	**53**		
Raspberry (mg/g)	-	-	-	-	-	Freeze dried fruit, 80% MeOH 0.5% AcOH. HPLC-ESI-MS/MS; HPLC-DAD. DPPH, ABTS, FRAP.	[63]
Bilberry (mg/g)	-	0.8	-	-	-		
Blackberry (mg/g)	-	-	-	-	-		
Blueberry (mg/g)	-	-	-	-	-		
Blueberry Wild (mg/100 g FW)	-	-	34.7 - 131.5	4.9 - 20.7	-	Ground fruit (85:15 v/v, MeOH:HCl) RP-HPLC-DAD. DPPH, ABTS, FRAP,ORAC.	[47]
Blueberry monocultivar (mg/100 g FW)	-	-	25.1- 76.5	ND - 37.2	-		

Cranberry (mg/g)	-	-	-	-	-	Freeze dried fruit, 80% MeOH 0.5% AcOH. HPLC-ESI-MS/MS; HPLC-DAD. DPPH, ABTS, FRAP.	[63]
Crowberry (mg/g)	-	1.4	-	-	-		
Mulberry (mg/g)	-	-	-	-	-		
Blackcurrant (mg/g)	-	-	-	-	-		
Blackcurrant Commercial (%)	-	-	-	-	-	Fresh berries extracted with 95% (v/v) ethanol acidified with 0.1N HCl. HPLC–UV–MS	[50]
Redcurrant (mg/g)	-	-	-	-	-	Freeze dried fruit, 80% MeOH 0.5% AcOH. HPLC-ESI-MS/MS; HPLC-DAD. DPPH, ABTS, FRAP.	[63]
Strawberry (mg/g)	-	-	-	-	-		
Pomegranate juice (mg/L)	-	-	-	-	-	Juice clarified 5 mL 15% gelatine HPLC, TEAC	[46]
Green rye grains (mg/Kg DW)	-	-	-	-	-	Dried and ground grain 70% EtOH and 1% (w/v) citric acid, purification, HPLC-ESI-MS	[64]
Violet rye grains (mg/Kg DW)	-	-	-	-	-		
Red Chicory (mg/100 g FW)	-	-	-	-	-	Ground sample MeOH and 0.03% HCl) HPLC-PDA/-ESI+-MS. ABTS, CRUPAC, DPPH	[42]
Red Onion (mg/100 g FW)	-	-	-	-	-		
Red onion (mg/100 g DW)	0.1 - 0.4	-	-	-	-	Freeze powder sample MeOH 0.1% HCl, UPLC, LC–DAD–ESI–MS. DPPH, FRAP, ORAC	[39]
Eggplant (mg/100 g FW)	-	-	-	-	-	Ground sample MeOH and 0.03% HCl) HPLC-PDA/-ESI+-MS. ABTS, CRUPAC, DPPH	[42]
Eggplant Black Beauty (mg/100 g DW)	ND	-	-	-	-	Freeze powder sample MeOH 0.1% HCl, UPLC, LC–DAD–ESI–MS. DPPH, FRAP, ORAC	[39]
Purple sweet potato (mg/100 g FW)	-	-	-	-	-	Ground sample MeOH and 0.03% HCl) HPLC-PDA/-ESI+-MS. ABTS, CRUPAC, DPPH	[42]
Purple potato (mg/100 g DW)	2.7 - 4.9	-	-	-	-	Freeze powder sample MeOH 0.1% HCl, UPLC, LC–DAD–ESI–MS. DPPH, FRAP, ORAC	[39]
Red potato (mg/100 g DW)	ND	-	-	-	-		
Black carrot (mg/100 g FW)	-	-	-	-	-	Ground sample MeOH and 0.03% HCl) HPLC-PDA/-ESI+-MS. ABTS, CRUPAC, DPPH	[42]
Purple carrot (mg/100 g DW)	0.8 - 3.5	-	-	-	-	Freeze powder sample MeOH 0.1% HCl, UPLC, LC–DAD–ESI–MS. DPPH, FRAP, ORAC	[39]
Red cabbage Gario (mg/100 g DW)	8.2 - 8.3	-	-	-	-		
Purple cauliflower Graffitti (mg/100 g DW)	6.8 - 7.0	-	-	-	-		
Purple asparagus Albenga (mg/100 g DW)	ND	-	-	-	-		
Begonia, light red (µg/g)	-	-	-	ND	759.0 - 759.1	Maceration of flower (1:10) (80% MeOH, 19% H_2O and 1% HCl, v/v/v). HPLC-DAD DPPH, FRAP, ORAC	[65]
Torenia, purple (µg/g)	-	-	-	ND	ND		
Mini rose, dark red (µg/g)	-	-	-	ND	ND		
Clitoria, blue (µg/g)	-	-	-	ND	16.5 -16.6		
Mini daisy, white (µg/g)	-	-	-	ND	ND		
Tagete, orange (µg/g)	-	-	-	ND	ND		
Cosmos, yellow (µg/g)	-	-	-	3.0 - 3.1	ND		
Cravine, red wine (µg/g)	-	-	-	2274.3 - 2274.4	ND		

Note: ND, not detected; 49, Malvidin; 50, Malvidin 3-arabinoside; 51, Malvidin 3-galactoside; 52, Malvidin 3-glucoside; 53, Malvidin 3,5-diglucoside

Table S6. Natural source, type of extraction, chromatographic method and antioxidant assay applied to identify and quantify pelargonidin and its derivatives identified with codes.

	Pelargonidin and its derivatives				Extraction, identification, quantification and antioxidant activity	References
Code	54	55	56	57		
Raspberry (mg/g)	-	0.7	-	-	Freeze dried fruit, 80% MeOH 0.5% AcOH. HPLC-ESI-MS/MS; HPLC-DAD. DPPH, ABTS, FRAP.	[63]
Bilberry (mg/g)	-	-	-	-		
Blackberry (mg/g)	-	-	-	-		
Blueberry (mg/g)	-	-	-	-		

Blueberry Wild (mg/100 g FW)	-	-	-	-	Ground fruit (85:15 v/v, MeOH:HCl) RP-HPLC-DAD. DPPH, ABTS, FRAP,ORAC.	[47]
Blueberry monocultivar (mg/100 g FW)	-	-	-	-		
Cranberry (mg/g)	-	-	-	-	Freeze dried fruit, 80% MeOH 0.5% AcOH. HPLC-ESI-MS/MS; HPLC-DAD. DPPH, ABTS, FRAP.	[63]
Crowberry (mg/g)	-	-	-	-		
Mulberry (mg/g)	-	1.7	0.54	-		
Blackcurrant (mg/g)	-	-	-	-		
Blackcurrant Commercial (%)	-	-	-	-	Fresh berries extracted with 95% (v/v) ethanol acidified with 0.1N HCl. HPLC–UV–MS	[50]
Redcurrant (mg/g)	-	-	-	-	Freeze dried fruit, 80% MeOH 0.5% AcOH. HPLC-ESI-MS/MS; HPLC-DAD. DPPH, ABTS, FRAP.	[63]
Strawberry (mg/g)	-	5.1	-	-		
Pomegranate juice (mg/L)	-	10.2 - 11.0	-	10.7- 12.0	Juice clarified 5 mL 15% gelatine HPLC, TEAC	[46]
Green rye grains (mg/Kg DW)	-	-	-	-	Dried and ground grain 70% EtOH and 1% (w/v) citric acid, purification, HPLC-ESI-MS	[64]
Violet rye grains (mg/Kg DW)	-	-	-	-		
Red Chicory (mg/100 g FW)	-	-	-	-	Ground sample MeOH and 0.03% HCl) HPLC-PDA/-ESI+-MS. ABTS, CRUPAC, DPPH	[42]
Red Onion (mg/100 g FW)	-	-	-	-		
Red onion (mg/100 g DW)	ND	-	-	-	Freeze powder sample MeOH 0.1% HCl, UPLC, LC–DAD–ESI–MS. DPPH, FRAP, ORAC	[39]
Eggplant (mg/100 g FW)	-	-	-	-	Ground sample MeOH and 0.03% HCl) HPLC-PDA/-ESI+-MS. ABTS, CRUPAC, DPPH	[42]
Eggplant Black Beauty (mg/100 g DW)	ND	-	-	-	Freeze powder sample MeOH 0.1% HCl, UPLC, LC–DAD–ESI–MS. DPPH, FRAP, ORAC	[39]
Purple sweet potato (mg/100 g FW)	-	-	-	-	Ground sample MeOH and 0.03% HCl) HPLC-PDA/-ESI+-MS. ABTS, CRUPAC, DPPH	[42]
Purple potato (mg/100 g DW)	ND	-	-	-	Freeze powder sample MeOH 0.1% HCl, UPLC, LC–DAD–ESI–MS. DPPH, FRAP, ORAC	[39]
Red potato (mg/100 g DW)	16.0 - 32.7	-	-	-		
Black carrot (mg/100 g FW)	-	-	-	-	Ground sample MeOH and 0.03% HCl) HPLC-PDA/-ESI+-MS. ABTS, CRUPAC, DPPH	[42]
Purple carrot (mg/100 g DW)	0.4 - 0.6	-	-	-	Freeze powder sample MeOH 0.1% HCl, UPLC, LC–DAD–ESI–MS. DPPH, FRAP, ORAC	[39]
Red cabbage Gario (mg/100 g DW)	ND	-	-	-		
Purple cauliflower Graffitti (mg/100 g DW)	ND	-	-	-		
Purple asparagus Albenga (mg/100 g DW)	ND	-	-	-		
Begonia, light red (µg/g)	-	ND	-	ND	Maceration of flower (1:10) (80% MeOH, 19% H_2O and 1% HCl, v/v/v). HPLC-DAD DPPH, FRAP, ORAC	[65]
Torenia, purple (µg/g)	-	ND	-	ND		
Mini rose, dark red (µg/g)	-	ND	-	1631.0 - 1631.2		
Clitoria, blue (µg/g)	-	ND	-	ND		
Mini daisy, white (µg/g)	-	ND	-	ND		
Tagete, orange (µg/g)	-	3.7-3.8	-	ND		
Cosmos, yellow (µg/g)	-	ND	-	ND		
Cravine, red wine (µg/g)	-	ND	-	ND		

Note: ND, not detected; 54, Pelargonidin; 55, Pelargonidin 3-glucoside; 56, Pelargonidin 3-rutinoside; 57, Pelargonidin 3,5-diglucoside.

References

39. Li, H.; Deng, Z.; Zhu, H.; Hu, C.; Liu, R.; Young, J.C.; Tsao, R. Highly pigmented vegetables: Anthocyanin compositions and their role in antioxidant activities. *Food Res. Int*. **2012**, 46, 250–259.

42. Frond, A.D.; Iuhas, C.I.; Stirbu, I.; Leopold, L.; Socaci, S., Andreea, S.; Ayvaz, H.; Andreea, S.; Mihai, S.; Diaconeasa, Z.; et al. Phytochemical Characterization of Five Edible Purple-Reddish Vegetables: Anthocyanins, Flavonoids, and Phenolic Acid Derivatives. *Molecules* **2019**, 24, 1536.

46. Pala, C.U.; Toklucu, A.K. Effect of UV-C light on anthocyanin content and other quality parameters of pomegranate juice. *J. Food Compos. Anal.* **2011**, *24*, 790–795.

47. Bunea, A.; Rugina, D.O.; Pintea, A.M.; Conta, Z.; Bunea, C.I.; Socaciu, C. Comparative Polyphenolic Content and Antioxidant Activities of Some Wild and Cultivated Blueberries from Romania. Not Bot Horti Agrobo 2011, 39, 70–76.

50. Prior, R.L.; Go, L.; Wu, X.; Jacob, R.A.; Sotoudeh, G.; Kader, A.A.; Cook, R.A. Plasma antioxidant capacity changes following a meal as a measure of the ability of a food to alter in vivo antioxidant status. *J. Am. Coll. Nutr.* **2007**, *26*, 170–181.

63. Ogawa, K.; Sakakibara, H.; Iwata, R.; Ishii, T.; Sato, T.; Goda, T.; Shimoi, K.; Kumazawa, S. Anthocyanin Composition and Antioxidant Activity of the Crowberry (Empetrum nigrum) and Other Berries. J. Agric. Food Chem. 2008, 56, 4457–4462.

64. Zykin, P.A.; Andreeva, E.A.; Lykholay, A.N.; Tsvetkova, N.V.; Voylokov, A.V. Anthocyanin Composition and Content in Rye Plants with Different Grain Color. *Molecules* **2018**, *23*, 948.

65. de Morais, J.S.; Sant'Ana, A.S.; Dantas, A.M.; Silva, B.S.; Lima, M.S.; Borges, G.C., Magnani, M. Antioxidant activity and bioaccessibility of phenolic compounds in white, red, blue, purple, yellow and orange edible flowers through a simulated intestinal barrier. *Food Res. Int.* **2020**, 131, 109046.
